# Isolation and Characterization of Compounds from *Glycyrrhiza uralensis* as Therapeutic Agents for the Muscle Disorders

**DOI:** 10.3390/ijms22020876

**Published:** 2021-01-16

**Authors:** Eun Ju Lee, Sibhghatulla Shaikh, Khurshid Ahmad, Syed Sayeed Ahmad, Jeong Ho Lim, Soyoung Park, Hye Jin Yang, Won-Kyung Cho, Sang-Joon Park, Yong-Ho Lee, So-Young Park, Jin-Yeul Ma, Inho Choi

**Affiliations:** 1 Department of Medical Biotechnology, Yeungnam University, Gyeongsan 38541, Korea; gorapadoc0315@hanmail.net (E.J.L.); sibhghat.88@gmail.com (S.S.); ahmadkhursheed2008@gmail.com (K.A.); sayeedahmad4@gmail.com (S.S.A.); lim2249@naver.com (J.H.L.); 2 Research Institute of Cell Culture, Yeungnam University, Gyeongsan 38541, Korea; 3 Department of Physiology, College of Medicine, Yeungnam University, Daegu 42415, Korea; soyoung614@hanmail.net (S.P.); sypark@med.yu.ac.kr (S.-Y.P.); 4 Korean Medicine (KM) Application Center, Korea Institute of Oriental Medicine (KIOM), Daegu 701-300, Korea; hjyang@kiom.re.kr (H.J.Y.); wkcho@kiom.re.kr (W.-K.C.); jyma@kiom.re.kr (J.-Y.M.); 5 College of Veterinary Medicine, Kyungpook National University, Daegu 41566, Korea; psj26@knu.ac.kr; 6 Department of Biomedical Science, Daegu Catholic University, Gyeongsan 38430, Korea; ylee325@cu.ac.kr

**Keywords:** skeletal muscle, bioactive compounds, muscle regeneration, *Glycyrrhiza uralensis*

## Abstract

Skeletal muscle is the most abundant tissue and constitutes about 40% of total body mass. Herein, we report that crude water extract (CWE) of *G. uralensis* enhanced myoblast proliferation and differentiation. Pretreatment of mice with the CWE of *G. uralensis* prior to cardiotoxin-induced muscle injury was found to enhance muscle regeneration by inducing myogenic gene expression and downregulating myostatin expression. Furthermore, this extract reduced nitrotyrosine protein levels and atrophy-related gene expression. Of the five different fractions of the CWE of *G. uralensis* obtained, the ethyl acetate (EtOAc) fraction more significantly enhanced myoblast proliferation and differentiation than the other fractions. Ten bioactive compounds were isolated from the EtOAc fraction and characterized by GC-MS and NMR. Of these compounds (**4**-hydroxybenzoic acid, liquiritigenin, (*R*)-(-)-vestitol, isoliquiritigenin, medicarpin, tetrahydroxymethoxychalcone, licochalcone B, liquiritin, liquiritinapioside, and ononin), liquiritigenin, tetrahydroxymethoxychalcone, and licochalcone B were found to enhance myoblast proliferation and differentiation, and myofiber diameters in injured muscles were wider with the liquiritigenin than the non-treated one. Computational analysis showed these compounds are non-toxic and possess good drug-likeness properties. These findings suggest that *G. uralensis*-extracted components might be useful therapeutic agents for the management of muscle-associated diseases.

## 1. Introduction

Skeletal muscle is composed of multinucleated myofibers and accounts for about half of the total body weight [1]. Myogenesis refers to myofiber producing processes such as those that occur during embryogenesis, postnatal growth, and muscle tissue regeneration [2]. Myogenesis is an extremely coordinated process and is associated with various transcriptional networks, the important components of which are Pax7, Myf5, MYOD, myogenin (MYOG), and Myf6 [3]. Post-injury repair of skeletal muscle involves a series of complex events, which can be broadly categorized into three steps. The first involves the activation of quiescent muscle satellite cells (MSCs). In response to damage, this pool of cells with stemness character develops into committed myoblasts [4]. The next step involves myoblasts proliferation, which leads to the expansion of the progenitor cell population [5]. The final step involves withdrawal of proliferating myoblasts from the cell cycle and their terminal differentiation. Inter-myoblast fusion leads to renascent myofiber formation and the replacement of injured and dead myofibers [4,6]. MSC differentiation is essential for skeletal muscle regeneration and is regulated by interactions between MSCs and different extracellular matrix components and non-myogenic cells, such as macrophages and fibroblasts [7,8,9,10,11,12,13].

Life expectancy is increasing globally, and thus the incidences of age-associated chronic diseases and associated treatment-related costs are increasing. Sarcopenia is generally characterized by age-associated gradual decreases in skeletal muscle mass, strength, and function [14]. In addition, muscle regenerative ability also reduces progressively with age [15], and decline in the regenerative ability of progenitor cells during aging is attributed to age-related alterations in endocrine factors associated with myogenic potential [16]. However, regardless of the clinical consequence of sarcopenia, no drug treatment is currently available. At present, only exercise and protein nutrition are recommended to sustain muscle functions [14,17].

Various negative regulators increase protein degradation in muscular dystrophy and sarcopenia, and, of these, myostatin (MSTN) effectively inhibits muscle growth, and thus is regarded as a potential curative target for the treatment of muscle wasting associated diseases [18]. MSTN inhibits the expressions of the myogenic regulatory factors MYOD and Pax3, and, therefore, controls the proliferation and differentiation of myoblasts during myogenesis [19,20]. In addition, Atrogin1 and MurF1 appear to be critical for the increased proteolysis and ubiquitination of muscle proteins observed in various disorders [21]. Myosin light chains1 (MYL1) and MYL2 are degraded by MurF1 after denervation or during fasting conditions [22]. Nitrotyrosine is a reactive nitrogen species and a tyrosine nitration product, and its detection in protein has been described as a marker of cell damage, inflammation, and nitric oxide generation [23].

*Glycyrrhiza uralensis* is a familiar medicinal herb belonging to the family *Leguminoceae*. The main bioactive constituents of *G. uralensis* are triterpene saponins and different flavones such as licochalcone A, glycyrrhizic acid, isoliquiritigenin, liquiritigenin, and liquiritin which have been reported to exhibit different pharmacological activities, including anti-inflammatory, anti-allergic, antioxidant, antiulcer, hepatoprogenic, and neuroprotective activities [24,25,26]. Although the crude water extract (CWE) of *G. uralensis* and its bioactive constituents have been shown to exert various pharmacological activities, the effects of *G. uralensis* on myoblast proliferation and differentiation have not been evaluated. The present study was designated to investigate the effects of *G. uralensis* extracts on myoblast proliferation and differentiation to determine their potential beneficial effects in skeletal muscle.

## 2. Results

### 2.1. The CWE of G. uralensis Promoted Myoblast Proliferation and Differentiation

**Figure 1:**
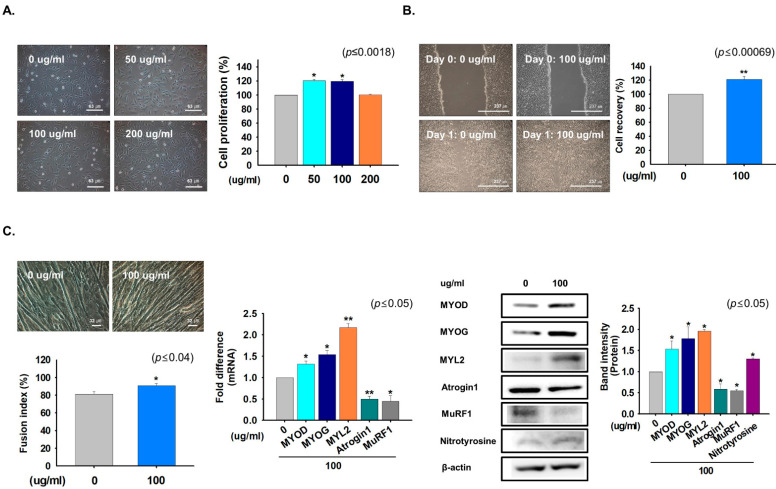
Effects of the crude-water extract of *G. uralensis* on cell proliferation and differentiation. (**A**) C2C12 cells were cultured in growth media supplemented with 0, 50, 100, or 200 µg/mL of *G. uralensis* CWE for one day. Cell proliferations in the presence of *G. uralensis* CWE as determined by MTT assay. (**B**) Scratch analysis was performed on ~100% confluent cells, incubated in growth medium containing 0 or 100 µg/mL of *G. uralensis* CWE for one day. Cell recovery was measured in treated and non-treated cells. (**C**) Cells were incubated with differentiation media supplemented with 100 µg/mL of *G. uralensis* CWE for four days. Myotube formation and fusion indices were determined by Giemsa staining, mRNA levels by real-time RT-PCR, and protein expressions by Western blotting. Non-treated cells were used as controls. Means ± SD (*n* = 6). * *p* ≤ 0.05, ** *p* ≤ 0.001.

The CWE of *G. uralensis* was added at different concentrations (0, 50, 100, or 200 µg/mL) to C2C12 cells cultured in growth media for one day. Cell proliferation was significantly increased by 50 and 100 µg/mL treatments versus non-treated controls (Figure 1A). In addition, expression of Ki67 (cellular marker for proliferation), CyclinA2 (regulator of cell cycle), and MSTN (inhibitor of myoblast cell proliferation) mRNA was analyzed in *G. uralensis* CWE treated cells (0 or 100 µg/mL for 1 day). Ki67 expression was significantly increased and MSTN expression was decreased with *G. uralensis* CWE treatment (Supplementary Figure S1). Next, scratch testing was performed on 100% confluent cells, which were incubated with 0 or 100 µg/mL of the CWE of *G. uralensis* for one day. Cell recoveries of treated cells (100 µg/mL) were better than those of non-treated controls (Figure 1B). 

In addition, cells were cultured in differentiation media supplemented with 100 µg/mL of *G. uralensis* for four days to determine its effects on myoblast differentiation. Treatment with the CWE of *G. uralensis* induced myotube formation and elevated the mRNA and protein expression of myogenic factors. However, the expressions of atrophy and protein degradation related marker genes (Atrogin1 and MurF1) were lower in cells treated with *G. uralensis* CWE than in non-treated controls (Figure 1C). These results show that *G. uralensis* CWE induced myoblast proliferation and differentiation by enhancing the expressions of myogenic genes and inhibiting atrophy-related genes expression.

### 2.2. The CWE of G. uralensis Enhanced Muscle Regeneration

**Figure 2:**
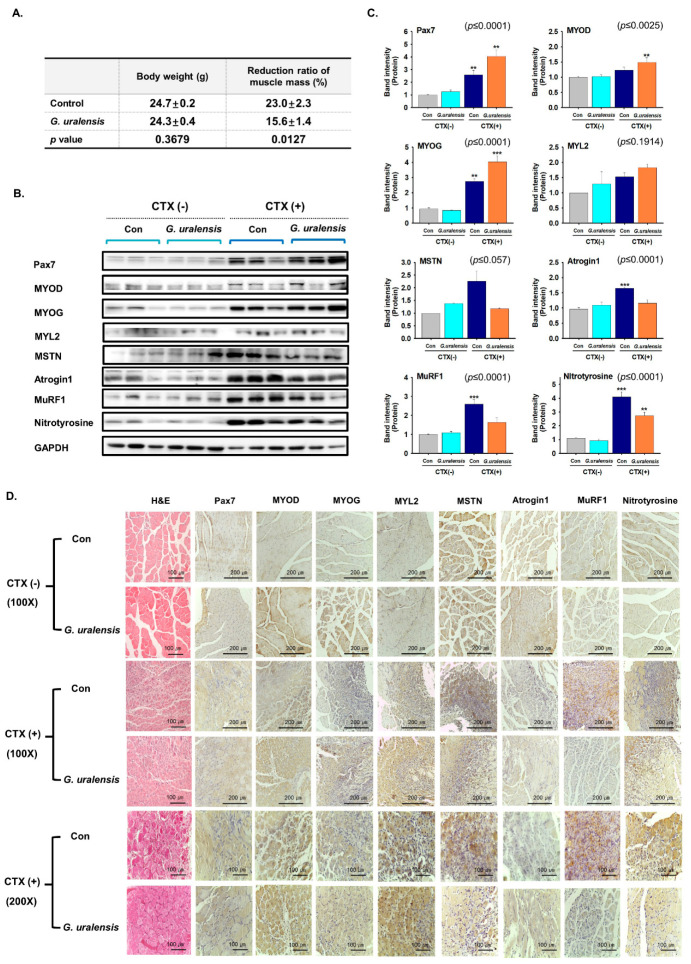
Muscle regeneration by the CWE of *G. uralensis*. Mice were orally administered *G. uralensis* CWE (100 mg/kg) daily for nine days from one day before CTX injection. Seven days after CTX injection, gastrocnemius muscle tissues were collected from control (intact or CTX injected animals) and *G. uralensis CWE* treated animals and from treatment naïve controls. (**A**) Body weights (g) and reductions in muscle mass (%). (**B**,**C**) Protein expression was determined by Western blotting and band intensities using Multi Gauge 3.0 software. (**D**) H&E staining and protein expression as determined by immunohistochemistry. Con indicates control. Means±SDs (*n* = 9). ** *p* ≤ 0.001, *** *p* ≤ 0.0001.

In order to investigate the effects of *G. uralensis* CWE on muscle regeneration, it was administered once a day for nine days from one day before CTX injection. Pre-treatment of *G. uralensis* CWE along with muscle injury can induce faster and stronger cellular signaling preferentially due to the effect of *G. uralensis* extract. Muscle tissues were collected and body weights (g), percentage muscle mass reduction (%), and myogenic and atrophy related protein expression were analyzed. *G. uralensis* CWE did not influence body weight but suppressed reductions in muscle mass as compared with non-treated muscles (Figure 2A). In addition, *G. uralensis* CWE enhanced the expressions of Pax7, MYOD, MYOG, and MYL2 and reduced those of MSTN, Atrogin1, and MuRF1 and nitrotyrosine levels in regenerated muscle tissues as compared with non-CTX injected muscle (Figure 2B–D). These observations show that *G. uralensis* CWE effectively promoted muscle regeneration by enhancing the expressions of myogenic genes and inhibiting those of atrophy-related genes. 

### 2.3. Fractions Derived from G. uralensis CWE Enhanced Myoblast Proliferation 

**Figure 3:**
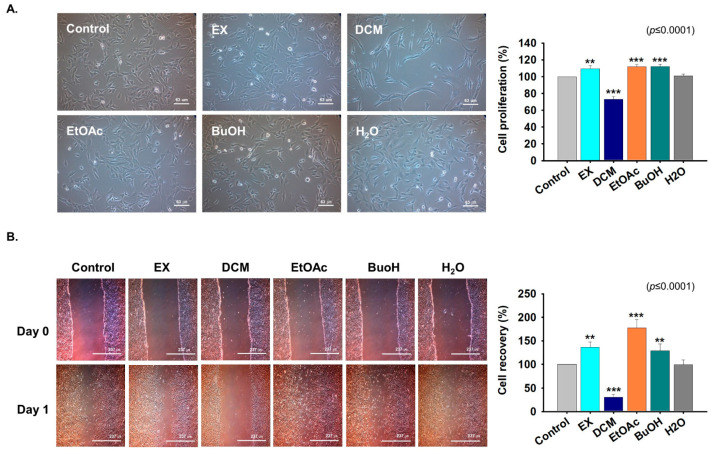
Cell proliferation in the presence of the five fractions of the CWE of *G. uralensis*. (**A**) C2C12 cells were cultured in growth media supplemented with the five different fractions (25 µg/mL) of *G. uralensis* CWE for one day. Cell proliferations after treatment with the EX, DCM, EtOAc, BuOH, or H_2_O fractions as determined by MTT assay. (**B**) When the cells were reached at 100% confluency, scratch was performed after incubation with each fraction for one day. Cell recoveries with the different *G. uralensis* versus non-treated cells. Means ± SD (*n* = 10). ** *p* ≤ 0.001, *** *p* ≤ 0.0001.

Five different fractions (water extracts; EX, Dichloromethane; DCM, Ethyl acetate; EtOAc, n-butanol; BuOH, and H_2_O; aqueous layer) from *G. uralensis* water extract were separated with different solvents (Supplementary Figure S2) and C2C12 cells were treated with each fraction for one day. Cell proliferation was significantly enhanced by the EX, EtOAc, and BuOH fractions, but decreased by the DCM fraction versus non-treated controls (Figure 3A). Scratch analysis was carried out on 100% confluent cells incubated with each of the five fractions for one day. Recovered area was larger in the sample treated with the Ex, EtOAc, and BuOH fractions respect to control (Figure 3B). These results show that the EX, EtOAc, and BuOH fractions of *G. uralensis* CWE enhance myoblast proliferation.

### 2.4. Effects of G. uralensis CWE Derived Fractions on Myoblast Differentiation

**Figure 4:**
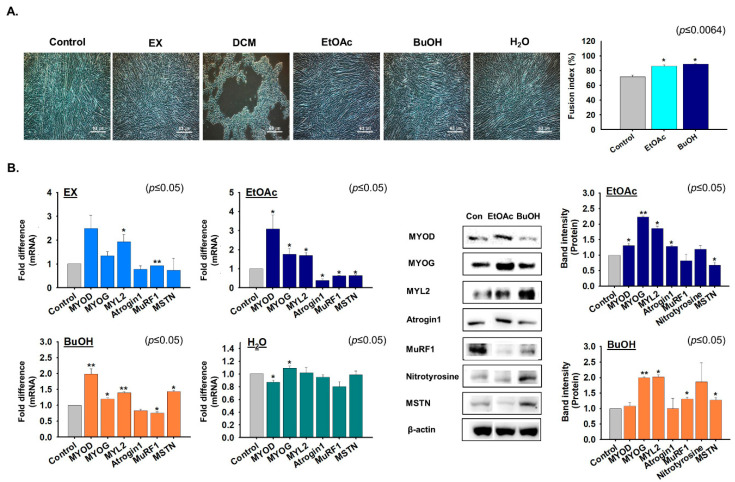
Cell differentiation in the presence of the five fractions of *G. uralensis CWE.* When C2C12 cells have researched 100% confluency, cells were incubated in differentiation media supplemented with 25 µg/mL of each of the five *G. uralensis* fractions (EX, DCM, EtOAc, BuOH, or H_2_O) for four days. (**A**) Myotube formation and fusion indices were assessed by Giemsa staining. (**B**) mRNA expression as determined by real time RT-PCR and protein expression by Western blot. Non-treated cells were used as controls. Means ± SD (*n* = 3). * *p* ≤ 0.05, ** *p* ≤ 0.001.

C2C12 cells were incubated in differentiation media supplemented with the EX, DCM, EtOAc, BuOH, or H_2_O fractions for four days to determine their effects on myoblast differentiation. Myotube formation was enhanced by the EtOAc and BuOH fractions versus non-treated cells. When cells were treated with DCM during differentiation, most cells detached from plates (Figure 4A). In addition, the expressions of myogenic marker genes were increased by EX (MYL2), EtOAc (MYOD, MYOG, and MYL2), BuOH (MYOD, MYOG, and MYL2), and H_2_O (MYOG) fractions. Furthermore, the expression of atrophy-related genes were significantly decreased by EX (MuRF1), EtOAc (Atrogin1 and MuRF1), and BuOH (MuRF1). Interestingly, MSTN and nitrotyrosine protein expression were lower in EtOAc fraction treated cells and higher in BuOH fraction treated cells than in controls (Figure 4B). In addition, protein expression in EtOAc or BuOH fraction treated cells were consistent with their mRNA expressions (Figure 4B). Following metabolite analysis, NH_3_ concentrations (a by-product of protein degradation) were lower in EtOAc fraction than in non-treated culture media (Supplementary Figure S3). These results suggest that the EtOAc fraction inhibited protein degeneration by inhibiting Atrogin1 expression. Taken together, these results suggest that *G. uralensis* derived EtOAc fraction enhanced myoblast differentiation by inducing the expression of myogenic marker genes and inhibiting those of MSTN and atrophy-related genes. 

### 2.5. G. uralensis-EtOAc Derived Compounds Increased Myoblast Proliferation and Differentiation

**Figure 5:**
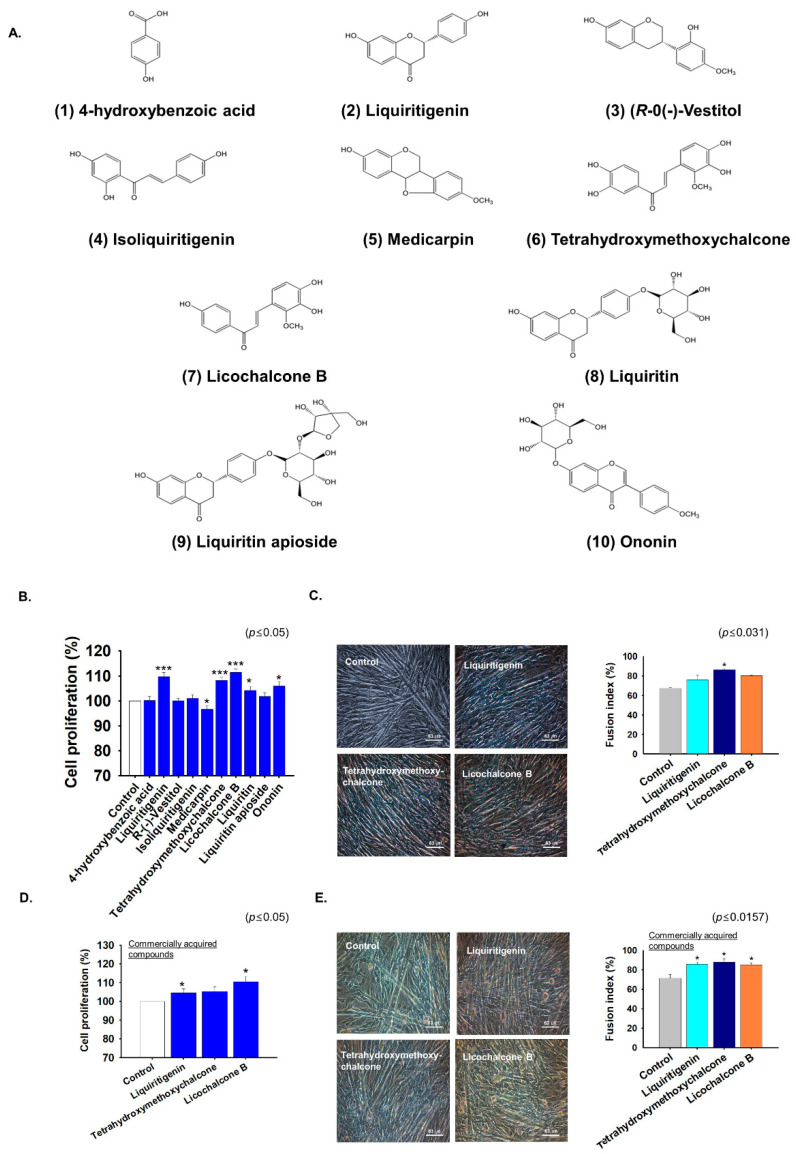
Cell proliferation and differentiation in the presence of single compounds present in the EtOAc fraction of the CWE of *G. uralensis*. (**A**) Structure of *G.* *uralensis*-EtOAc fraction derived single compounds. (**B**) C2C12 cells were incubated in growth media supplemented with either of the ten single compounds isolated from *G. uralensis*-EtOAc for 1 day. Cell proliferations in the presence of single compounds (0.25 ng/mL) determined by MTT assay. (**C**) When the cells have reached 100% confluency, cells were incubated in differentiation media supplemented with liquiritigenin, tetrahydroxymethoxychalcone, or licochalcone B (0.5 ng/mL) for four days. Myotube formation and fusion indices in the presence of the three compounds as determined by Giemsa staining. (**D**) Cell proliferation in growth media supplemented with liquiritigenin (0.25 ng/mL), tetrahydroxymethoxychalcone (0.25 ng/mL), or licochalcone B (1 ng/mL) as determined by MTT assay. (**E**) Cell differentiation in differentiation media supplemented with liquiritigenin, tetrahydroxymethoxychalcone, or licochalcone B at 0.25 ng/mL as determined by Giemsa staining. Non-treated cells were used as controls. Means ± SD (*n* = 14). * *p* ≤ 0.05, *** *p* ≤ 0.0001.

The EtOAc-soluble fraction of *G. uralensis* CWE, which significantly enhanced myoblast proliferation and differentiation, was selected for further investigation. Ten bioactive compounds were isolated from this fraction by GC-MS analysis, that is, one phenolic compound (**1**) and nine flavonoids (**2**–**10**). Their structures were determined by NMR as **4**-hydroxybenzoic acid (1) [27,28], liquiritigenin (2) [29,30], (*R*)-(-)-vestitol (3) [31], isoliquiritigenin (4) [32,33], medicarpin (5) [34], tetrahydroxymethoxychalcone (6) [35], licochalcone B (7) [36,37,38], liquiritin (8) [39,40], liquiritinapioside (9) [35,41], and ononin (10) [42,43] (Table 1 and Figure 5A).

To investigate the effects of these ten single compounds on cell proliferation, each was added at 0.5 ng/mL to C2C12 cells in growth medium and incubated for 1 day. Cell proliferation was increased by liquiritigenin (10%), tetrahydroxymethoxychalcone (8%), licochalcone B (11%), liquiritin (4%), liquiritinapioside (2%), and ononin (6%) versus non-treated controls (Figure 5B). 

Next, C2C12 cells were cultured in differentiation media supplemented with liquiritigenin, tetrahydroxymethoxychalcone, or licochalcone B for four days to determine their effects on myoblast differentiation. Myotube formation was increased by tetrahydroxymethoxychalcone versus non-treated controls (Figure 5C). Three compounds (liquiritigenin, tetrahydroxymethoxychalcone, or licochalcone B) were commercially available. After acquiring these compounds, cell proliferation and differentiation were analyzed in proliferation medium for one day or differentiation medium for four days. Cell proliferation was significantly increased by liquiritigenin (0.25 ng/mL; 5%) and by licochalcone B (1 ng/mL; 11%) (Figure 5D), and, interestingly, myogenic differentiation was also increased by each of the three commercial standards (Figure 5E). These results show that liquiritigenin, tetrahydroxymethoxychalcone, or licochalcone B enhance myoblast proliferation and differentiation. 

### 2.6. Liquiritigenin Enhanced Muscle Regeneration

**Figure 6:**
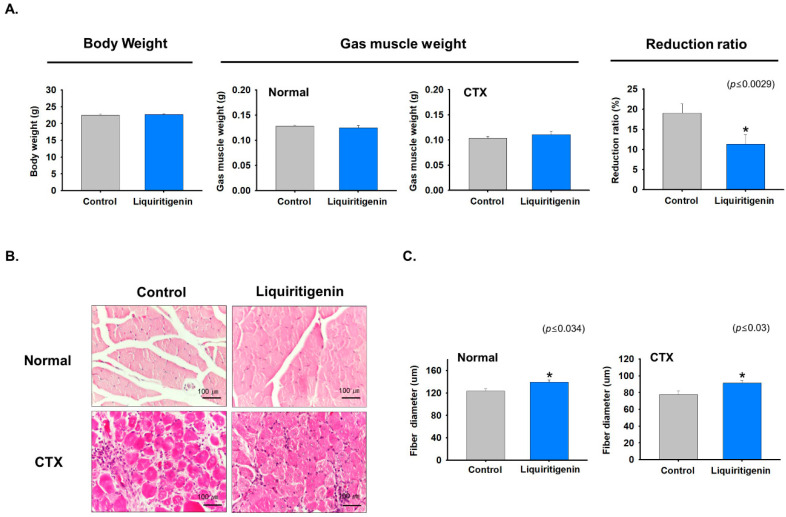
Muscle regenerative effect of liquiritigenin. Mice were orally administered liquiritigenin once a day for nine days from one day before CTX injection. Seven days after CTX injection, muscle tissues were collected from CTX injected and/or liquiritigenin treated mice and treatment naïve controls. (**A**) Body weights (g), gastrocnemius muscle weights (g), and reductions in muscle mass (%). (**B**) H&E stained muscle fibers of CTX injected and/or liquiritigenin treated and treatment naïve controls. (**C**) Muscle fiber diameters of CTX injected and/or liquiritigenin treated and treatment naïve controls determined using Image J. Means ± SD (*n* = 10). * *p* ≤ 0.05. Intact /Control (no CTX injection + no treatment), Intact /Liquiritigenin (no CTX injection + Liquiritigenin treatment), CTX/Control (CTX injection + no treatment), CTX/Liquiritigenin (CTX injection + Liquiritigenin treatment).

In order to investigate the effects of liquiritigenin on muscle regeneration, mice were orally administered liquiritigenin daily for nine days from one day before CTX injection. Muscle tissues were collected and body weights (g), gastrocnemius muscle weights (g), and muscle mass reductions (%) were analyzed. Body and gastrocnemius muscle weights were similar for liquiritigenin treated and non-treated controls. However, muscle mass reductions in liquiritigenin treated mice were less than in non-treated controls (Figure 6A). In addition, muscle fiber size is increased after liquiritigenin treatment, both in non-injected and in CTX-injected regenerating muscles compared to non-treated ones (Figure 6B,C). These observations showed that liquiritigenin enhanced muscle regeneration.

### 2.7. FAF-Drugs 4 Based Analysis of Liquiritigenin, Tetrahydroxymethoxychalcone, and Licochalcone B

**Figure 7:**
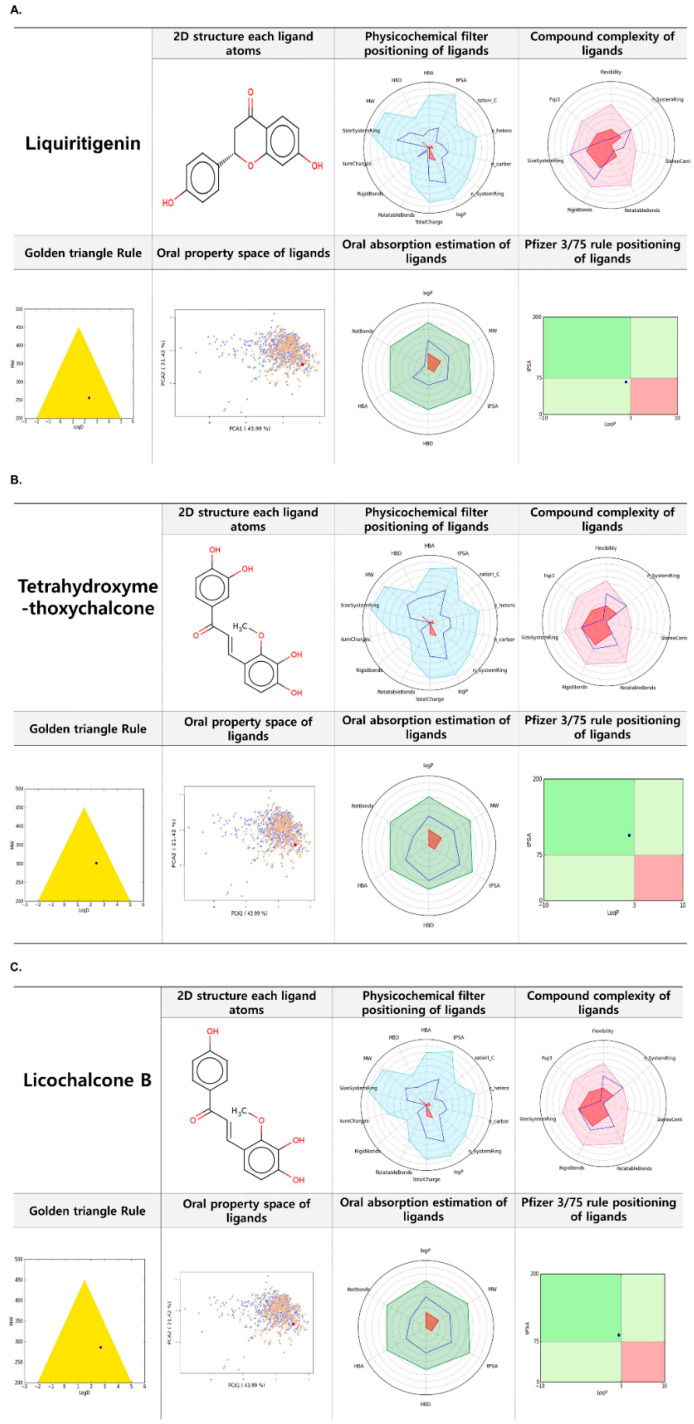
FAF-Drugs4 results for the Top 3 compounds and their respective ADME properties. (**A**) liquiritigenin, (**B**) tetrahydroxymethoxychalcone, (**C**) licochalcone B.

*In silico* predictions of the biological properties and toxicities of chemical compounds provide a rapid, dependable means of assessment before further bench-work is conducted, and physicochemical and ADMET properties are essential considerations for any candidate drug. Liquiritigenin, tetrahydroxymethoxychalcone, and licochalcone B were found to be suitable for FAF-Drug4 analysis in terms of molecular complexity, number of aromatic rings, sp3 hybridized (Fsp3) carbon fractions, and ADMET properties [44], which indicated these compounds are non-toxic and might be therapeutically useful (Figure 7A–C).

## 3. Discussion

Population aging is a comparatively recent global phenomenon, hence greater awareness is needed to ensure healthy aging and quality of life. Physical exercise and a high protein diet are known to contribute to the maintenance of muscle function in the elderly, but these approaches are restricted to healthy subjects rather than those suffering from disease or immobility [45]. Hence, the present study was undertaken to determine whether *G. uralensis* has the potential to promote myogenesis and muscle functions. In this study, we found that extracts of *G. uralensis* enhanced the proliferation and differentiation of C2C12 myoblasts, and that pre-treatment with *G. uralensis* extracts enhanced muscle regenerative ability in a CTX-induced mouse model of muscle injury. In a previous study, Hachimijiogan (a Japanese herbal medicine) has found to enhance C2C12 myoblast proliferation but not to affect or induce their differentiation [46]. In contrast, we found that the *G. uralensis* extract promoted the proliferation and differentiation of C2C12 myoblasts.

Furthermore, *G. uralensis* extract was found to promote myogenesis by upregulating the expressions of myogenic marker genes (MYOD, MYOG, and MYL2), which is important, as MYOD and MYOG are recognized myogenic regulatory factors that perform essential functions during myogenesis [47]. In addition, following muscle injury, pretreatment with *G. uralensis* extract enhanced muscle regeneration by inducing the expression of myogenic mRNA and proteins, which pointed the muscle regenerative ability of *G. uralensis*. 

Cachexia, unlike sarcopenia, is an intricate metabolic syndrome primarily characterized by extreme loss of muscle mass with or without fat loss. Abnormal metabolism is a major characteristic of cachexia, and several metabolic pathways in different tissues are known to be disturbed in this condition [48]. Cachexia is related to multiple chronic diseases, most commonly cancer. Effective therapeutic strategies for cancer-associated cachexia include a combination of multiple approaches aimed at stabilizing metabolic alterations [48,49]. MSTN is an important target because it is overexpressed in many cachectic disorders [50]. We previously found that curcumin and gingerol potently inhibit MSTN and help suppress the expressions of advanced glycation end products and that of their receptor RAGE [51], which is also viewed as a potential therapeutic target for cancer cachexia [52]. Here, we report that the EtOAc derived fractions of *G. uralensis* reduced the expressions of MSTN, Atrogin1, and MurF1, which suggests it has therapeutic potential against muscle wasting in muscular dystrophy.

We found that *G. uralensis* extract reduced the level of nitrotyrosine, which suggests that the extract has anti-inflammatory properties. Licorice extract has been reported to reduce proinflammatory cytokine levels in serum [53], and studies on bioactive compounds in *G. uralensis* have shown they also exhibit anti-inflammatory effects. For instance, Su et al. [54] concluded that the antidepressant and antianxiety effects of liquiritigenin were associated with its anti-inflammatory effect, as liquiritigenin pretreatment reduced pro-inflammatory cytokine (IL-6 and TNF-α) levels. In another study, isoliquiritigenin was observed to exert anti-inflammatory effects by reducing lipopolysaccharide-stimulated inducible nitric oxide synthase (iNOS) and cyclooxygenase-2 expressions by inhibiting the activation of nuclear factor-kappa B in RAW264.7 macrophages [55]. Furthermore, in mice, licochalcone A in diet decreased the expressions of iNOS and cyclooxygenase-2 and reduced proinflammatory cytokine levels in colon tissues [56]. All of these studies and the present study indicate *G. uralensis* has anti-inflammatory properties. 

In the present study, *G. uralensis* CWE enhanced the proliferation and differentiation of myoblast cells and muscle regeneration, and the EtOAc-fraction of *G. uralensis* CWE increased proliferation and differentiation significantly more than the other fractions. Therefore, we subjected the EtOAc-soluble fraction to further study to identify its bioactive constituents and determine their effects on myoblast proliferation and differentiation. Ten bioactive compounds were isolated from the EtOAc fraction and characterized by GC-MS and NMR. This analysis showed these compounds to be a phenolic and nine flavonoid compounds, and, among these, liquiritigenin, tetrahydroxymethoxychalcone, and licochalcone B were found to enhance myoblast proliferation and differentiation.

On a cautionary note, the presence of glycyrrhizin, a major active constituent of *G. uralensis*, suggests that excessive consumption of *G. uralensis* might have a toxic effect [57]. However, it should be emphasized that the compounds isolated from the EtOAc-fraction of *G. uralensis* that did not contain glycyrrhizin were found to be non-toxic by in silico analysis. Furthermore, myoblast proliferation and differentiation were increased by commercially procured liquiritigenin, tetrahydroxymethoxychalcone, and licochalcone B, and muscle regeneration was enhanced by orally administered liquiritigenin as compared with non-treated controls, which suggested that it plays important roles in myogenesis and muscle regeneration.

## 4. Materials and Methods

### 4.1. General Experimental Procedures

A BRUKER AVANCE III HD 600 unit (Bruker Biospin GmbH, Karlsruhe, Germany) was used to record ^1^H- and ^13^C NMR spectra at 600 and 150 MHz using tetramethylsilane as the internal standard. Medium-pressure liquid chromatography (MPLC) was carried out using an Isolera One machine (Biotage, Uppsala, Sweden) equipped with SNAP KP-SIL and SNAP Ultra C_18_ cartridges. Silica gel (Kieselgel 60, 70–230, and 230–400 mesh, Merck, Darmstadt, Germany). Column chromatography was performed using YMC C_18_ resins and thin-layer chromatography (TLC) was carried out using pre-coated silica gel 60 F_254_ and RP-18 F_254S_ plates (0.25 mm, Merck, Darmstadt, Germany). Plates were treated with 10% H_2_SO_4_ and visualized under UV light (254 and 365 nm).

### 4.2. Plant Material and Preparation of G. uralensis Water Extract (CWE)

The dried roots of *G. uralensis Fischer* were procured from the Kwangmyeong Herbal Store (KM Herb Co., Ltd., Busan, Korea). All voucher specimens were deposited in an herbal bank at the KM Application Center, Korea Institute of Oriental Medicine. To prepare a water extract, dried roots of *G. uralensis* (50 g) were added to 1000 mL distilled water and extracted by heating at 115 °C for 3 h. The extract obtained was filtered using standard testing sieves (150 μm) and freeze-dried. The lyophilized extract powder was then dissolved in tertiary distilled water and left at 4 °C for 24 h, centrifuged at 5000× *g* for 5 min, transferred to new tubes, and stored at −20 °C.

### 4.3. Extraction and Isolation

Dried roots of *G. uralensis* (4.2 kg) were extracted with water (fractionation for bioassay) or 100% methanol (MeOH, fractionation for isolation) under reflux three times (each for 15 L) (Supplementary Figure S2). The extract was then suspended in distilled water and partitioned versus dichloromethane (DCN; CH_2_Cl_2_) to obtain the crude water extract. Removal of the CH_2_Cl_2_ and partitioning the water fraction with ethyl acetate (EtOAc) followed by EtOAc evaporation (under reduced pressure) at 45 °C yielded the EtOAc extract (137.0 g). This extract was then subjected to silica gel column chromatography using hexane-EtOAc-MeOH (5.5:1:0.1, 3:1:0.1, *v/v*), CHCl_3_-Acetone-MeOH (3:1:0.1, *v/v*), and CHCl_3_-MeOH-H_2_O (5:1:0.1, 3:1:0.1, *v/v*) gradients to obtain 5 fractions (Fr. E1-E5) and a semi-crystalline solid (Fr. EC). Fraction E2 (4.9 g) was separated using a MeOH-Water (34–75% MeOH, *v/v*) gradient by MPLC using a SNAP Ultra C18 cartridge to give 16 fractions (Fr. E2A-E2P), which included compound **1** (23.9 mg). Fraction E2C (243.5 mg) was separated by silica gel column chromatography using hexane-EtOAc-MeOH (3:1:0.1, *v/v*) as eluent to give compound **2** (108.5 mg). Fraction E2D (420.3 mg) was isolated using a hexane-EtOAc-MeOH (17–25% EtOAc-MeOH (1:0.1), *v/v*) gradient by MPLC using a SNAP KP-SIL cartridge to give compounds **3** (51.7 mg) and 4 (15.4 mg). Fraction E2F (95.1 mg) was separated using a hexane-EtOAc-MeOH (11–22% EtOAc-MeOH (1:0.1), *v/v*) gradient by MPLC using a SNAP KP-SIL cartridge to give compound **5** (11.2 mg). Fraction E3 (41.6 g) was separated using a MeOH-Water (33–80% MeOH, *v/v*) gradient by MPLC using a SNAP Ultra C18 cartridge to give 15 fractions (Fr. E3A-E3O). Fraction E3E (140.0 mg) was isolated with a hexane-EtOAc-MeOH (28–36% EtOAc-MeOH (1:0.1), *v/v*) gradient by MPLC using SNAP KP-SIL cartridge to give compound **6** (35.2 mg). Fraction E3F (410.0 mg) was isolated using a hexane-EtOAc-MeOH (20–34% EtOAc-MeOH (1:0.1), *v/v*) gradient by MPLC using a SNAP KP-SIL cartridge to give 5 fractions (Fr. E3FA-E3FE). Fraction E3FB (17.5 mg) was separated by TLC (silica gel 60 F254, hexane-EtOAc-MeOH, 1:1:0.2, *v/v*) to give compound **7** (5.6 mg). Fraction E5 (20.4 g) was isolated using a MeOH-Water (20–44% MeOH, *v/v*) gradient by MPLC using a SNAP Ultra C18 cartridge to give 9 fractions (Fr. E5A-E5I). Fraction E5B-D was combined and re-chromatographed using a CHCl3-MeOH-H2O (12–20% MeOH-H2O (1:0.1), *v/v*) gradient by MPLC using a SNAP KP- SIL cartridge to give 5 fractions (Fr. E5BA-E5BE). Fraction E5BA was obtained as a semi-crystalline solid and recrystallization from MeOH to give compound **8** (969.0 mg). Fractions E5BB-C were combined and subjected to silica gel column chromatography using CHCl3-MeOH-H2O (7:1:0.05, 6:1:0.05 and MeOH, *v/v*) gradient to give 6 fractions (Fr. E5BBA-E5BBF). Fraction E5BBD (220.0 mg) was isolated using an EtOAc-MeOH-H2O (4–10% MeOH-H2O (1:0.1), *v/v*) gradient by MPLC using a SNAP KP-SIL cartridge to provide compound **9** (113.5 mg). Fraction EC (3.0 g) was separated with a CHCl3-MeOH-H_2_O (12–16% MeOH-H2O (1:0.1) and 100% Acetone, *v/v*) gradient by MPLC using a SNAP KP- SIL cartridge to give one fraction, which was recrystallized from MeOH to give compound **10** (22.2 mg). Their structures were elucidated on the basis of obtained spectroscopic data and comparison of NMR spectral data with previously reported data.

**4**-Hydroxybenzoic acid (1): White powder; C_7_H_6_O_3_; ^1^H NMR (600 MHz, MeOD-d_4_) δ 7.67 (2H, d, J = 7.2 Hz, H-2, 6), 6.61 (2H, d, J = 7.2 Hz, H-3, 5). ^13^C NMR (150 MHz, MeOD-d_4_) δ 170.1 (C=O), 163.3 (C-4), 132.9 (C-2, 6), 122.7 (C-1), 116.0 (C-3, 5) (Supplementary Figures S4 and S5) [27,28]. 

Liquiritigenin (2): Colorless needle crystals; C_15_H_12_O_4_; ^1^H NMR (600 MHz, MeOD-d_4_) δ 7.68 (1H, d, J = 8.5 Hz, H-5), 7.28 (2H, d, J = 7.6 Hz, H-2′, 6′), 6.77 (2H, d, J = 7.8 Hz, H-3′, 5′), 6.45 (1H, d, J = 6.5 Hz, H-6), 6.31 (1H, s, H-8), 5.32 (1H, d, J = 12.6 Hz, H-2), 3.00 (1H, t, J = 15.0 Hz, H-3a), 2.64 (1H, d, J = 16.8 Hz, H-3b). ^13^C NMR (150 MHz, MeOD-d_4_) δ 192.1 (C=O), 165.3 (C-7), 164.1 (C-9), 157.5 (C-4′), 129.9 (C-1′), 128.4 (C-5), 127.6 (C-2′, 6′), 114.9 (C-3′, 5′), 113.5 (C-10), 110.3 (C-6), 102.4 (C-8), 79.6 (C-2), 43.5 (C-3) (Supplementary Figures S6 and S7) [29,30].

(*R*)-(-)-Vestitol (3): Colorless crystals; C_16_H_16_O_4_; ^1^H NMR (600 MHz, Acetone-d_6_) δ 7.05 (1H, d, J = 7.7 Hz, H-6′), 6.89 (1H, d, J = 7.1 Hz, H-5), 6.50 (1H, s, H-3′), 6.42 (1H, d, J = 6.1 Hz, H-5′), 6.36 (1H, d, J = 5.7 Hz, H-6), 6.28 (1H, s, H-8), 4.23 (1H, d, J = 6.7 Hz, H-2), 3.98 (1H, t, J = 9.5 Hz, H-2), 3.72 (3H, s, -OCH3), 3.47 (1H, m, H-3), 2.99 (1H, m, H-4), 2.82 (1H, d, J = 14.8 Hz, H-4). ^13^C NMR (150 MHz, Acetone-d_6_) δ 160.3 (C-4′), 157.4 (C-7), 156.6 (C-2′), 156.1 (C-9), 131.0 (C-5), 128.7 (C-6′), 120.9 (C-1′), 114.3 (C-10), 108.7 (C-6), 105.6 (C-5′), 103.6 (C-8), 102.4 (C-3′), 70.4 (C-2), 55.3 (-OCH3), 32.6 (C-3), 31.0 (C-4) (Supplementary Figures S8 and S9) [31].

Isoliquiritigenin (4):Yellow crystals; C_15_H_12_O_4_; ^1^H NMR (600 MHz, MeOD-d_4_) δ 7.90 (1H, d, J = 8.2 Hz, H-6′), 7.73 (1H, d, J = 15.2 Hz, H-β), 7.56 (3H, s, H-2, 6, α), 6.79 (2H, d, J = 8.3 Hz, H-3, 5), 6.36 (1H, dd, J = 8.6, 1.6 Hz, H-5′), 6.24 (1H, s, H-3′). ^13^C NMR (150 MHz, MeOD-d_4_) δ 193.5 (C=O), 167.5 (C-4′), 166.3 (C- 2′), 161.5 (C-4), 145.6 (C-β), 133.3 (C-6′), 131.8 (C-2, 6), 127.8 (C-1), 118.2 (C-α), 116.9 (C-3, 5), 114.7 (C-1′), 109.1 (C-5′), 103.8 (C- 3′) (Supplementary Figures S10 and S11) [32,33].

Medicarpin (5): White amorphous powder; C_16_H_14_O_4_;^1^H NMR (600 MHz, CDCl_3_) δ 7.39 (1H, d, J = 7.5 Hz, H-1), 7.13 (1H, d, J = 7.1 Hz, H-7), 6.55 (1H, d, J = 6.0 Hz, H-2), 6.45 (2H, s, H-8, -10), 6.42 (1H, s, H-4), 5.50 (1H, d, J = 5.8 Hz, H-11a), 4.24 (1H, d, J = 5.4 Hz, H-6_eq_), 3.77 (3H, s, -OCH_3_), 3.62 (1H, br t, J = 10.4 Hz, H-6_ax_), 3.54 (1H, d, J = 4.3 Hz, H-6a_)._^13^C NMR (150 MHz, CDCl_3_) δ 161.2 (C-9), 160.7 (C-10a), 157.1 (C-3), 156.8 (C-4a), 132.3 (C-1), 124.9 (C-7), 119.2 (C-6b), 112.8 (C-11b), 109.9 (C-2), 106.5 (C-8), 103.8 (C-4), 97.0 (C-10), 78.6 (C-11a), 66.6 (C-6), 55.6 (-OCH_3_), 39.6 (C-6a) (Supplementary Figures S12 and S13) [34].

Tetrahydroxy-methoxy-chalcone (6): Yellow needles; C_16_H_14_O_6_;^1^H NMR (600 MHz, MeOD-d_4_) δ 7.89 (1H, d, J = 15.6 Hz, H-β), 7.55 (1H, d, J = 15.6 Hz, H-α), 7.48 (1H, d, J = 6.7 Hz, H-2′), 7.45 (1H, s, H-6′), 7.14 (1H, d, J = 7.9 Hz, H-6), 6.82 (1H, d, J = 7.5 Hz, H-5′), 6.59 (1H, d, J = 7.9 Hz, H-5), 3.78 (3H, s, -OCH_3_). ^13^C NMR (150 MHz, MeOD-d_4_) δ 191.4 (C=O), 152.1 (C-4′), 150.7 (C-4), 149.9 (C-2), 146.5 (C-3′), 140.9 (C-3), 139.6 (C-β), 131.8 (C-1′), 123.4 (C-6′), 121.4 (C-1), 120.5 (C-α), 120.3 (C-6), 116.3 (C-2′), 115.9 (C-5′), 112.7 (C-5), 61.7 (-OCH_3_) (Supplementary Figures S14 and S15) [35].

Licochalcone B (7): Yellow needles; C_16_H_14_O_5_;^1^H NMR (600 MHz, MeOD-d_4_) δ 7.97–7.91 (3H, m, H-2′, 6′, β), 7.61 (1H, d, J = 15.7 Hz, H-α), 7.19 (1H, d, J = 7.9 Hz, H-6), 6.85 (2H, d, J = 6.9 Hz, H-3′, 5′), 6.61 (1H, d, J = 7.5 Hz, H-5), 3.81 (3H, s, -OCH_3_). ^13^C NMR (150 MHz, MeOD-d_4_) δ 191.3 (C=O), 163.9 (C-4′), 151.0 (C-4), 149.9 (C-2), 141.0 (C-β), 139.7 (C-3), 132.2 (C-2′, 6′), 131.1 (C-1′), 121.3 (C-1), 120.4 (C-α), 120.3 (C-6), 116.4 (C-3′, 5′), 112.7 (C-5), 61.7 (-OCH_3_) (Supplementary Figures S16 and S17) [36,37,38].

Liquiritin (8): White powder; C_21_H_22_O_9_;^1^H NMR (600 MHz, DMSO-d_6_) δ 7.65 (1H, d, J = 7.6 Hz, H-5), 7.44 (2H, s, H-2′, 6′), 7.06 (2H, s, H-3′, 5′), 6.51 (1H, s, H-6), 6.35 (1H, s, H-8), 4.88 (1H, s, H-1″), 3.68 (1H, s, H-6″), 3.10–3.50 (6H, m, H-2″, 3″, 4″, 5″, 6″, 3), 2.67 (1H, d, J = 15.2 Hz, H-3). ^13^C NMR (150 MHz, DMSO-d_6_) δ 190.4 (C=O), 165.1 (C-7), 163.5 (C-9), 157.9 (C-4′), 132.8 (C-1′), 128.8 (C-5), 128.4 (C-2′, 6′), 116.6 (C-3′, 5′), 114.0 (C-10), 111.0 (C-6), 103.0 (C-8), 100.7 (C-1″), 79.1 (C-2), 77.5 (C-5″), 77.0 (C-3″), 73.6 (C-2″), 70.1 (C-4″), 61.1 (C-6″), 43.6 (C-3) (Supplementary Figures S18 and S19) [39,40].

Liquiritinapioside (9): Yellow powder; C_26_H_30_O_13_; ^1^H NMR (600 MHz, MeOD-d_4_) δ 7.71 (1H, d, J = 8.3 Hz, H-5), 7.41 (2H, d, J = 6.9 Hz, H-2′, 6′), 7.09 (2H, s, H-3′, 5′), 6.48 (1H, d, J = 6.4 Hz, H-6), 6.34 (1H, s, H-8), 5.44 (1H, br s, H-1‴), 5.40 (1H, s, H-2), 4.98 (1H, d, J = 7.0 Hz, H-1″), 3.01 (1H, t, J = 14.5 Hz, H-3), 2.70 (1H, t, J = 16.8 Hz, H-3). ^13^C NMR (150 MHz, MeOD-d_4_) δ 193.2 (C=O), 166.8 (C-7), 165.4 (C-9), 159.1 (C-4′), 134.3 (C-1′), 129.8 (C-5), 128.8 (C-2′, 6′), 117.5 (C-3′, 5′), 115.0 (C-10), 111.8 (C-6), 110.7 (C-1‴), 103.8 (C-8), 100.7 (C-1″), 80.7 (C-3‴), 80.7 (C-2), 78.6 (C-2″), 78.5 (C-3″), 78.0 (C-2‴), 78.0 (C-5″), 75.4 (C-4‴), 71.3 (C-4″), 66.0 (C-5‴), 62.4 (C-6″), 44.9 (C-3) (Supplementary Figures S20 and S21) [35,41].

Ononin (10): White powder; C_22_H_22_O_9_; ^1^H NMR (600 MHz, DMSO-d_6_) δ 8.43 (1H, s, H-2), 8.05 (1H, d, J = 8.5 Hz, H-5), 7.53 (2H, d, J = 7.5 Hz, H-2′, 6′), 7.24 (1H, s, H-8), 7.15 (1H, d, J = 8.1 Hz, H-6), 6.99 (2H, d, J = 7.6 Hz, H-3′, 5′), 5.12 (1H, d, J = 7.8 Hz, H-1″), 3.78 (3H, s, -OCH_3_), 3.73 (1H, m, C-6″), 3.19–3.48 (5H, m, H-2″, 3″, 4″, 5″, 6″). ^13^C NMR (150 MHz, DMSO-d_6_) δ 175.1 (C-4), 161.9 (C-7), 159.4 (C-4′), 157.5 (C-9), 154.1 (C-2), 130.5 (C-2′, 6′), 127.4 (C-5), 124.4 (C-1′), 123.8 (C-3), 118.9 (C-10), 116.0 (C-6), 114.0 (C-3′, 5′), 103.8 (C-8), 100.4 (C-1″), 77.6 (C-5″), 76.9 (C-3″), 735. (C-2″), 70.7 (C-4″), 61.0 (C-6″), 55.6 (-OCH_3_) (Supplementary Figures S22 and S23) [42,43].

### 4.4. Animal Experiment

C57BL/6 male mice were purchased from KOATECH (Seoul, Korea) and kept per cage in a temperature-controlled room under a 12 h light/12 h dark cycle. Animals were provided with standard rodent chow containing 6.2% (wt/wt) total fat (Envigo, Indianapolis, IN, USA) and water. All experiments conformed to the guidelines and regulations issued by the Animal Care Committee of our institute, and all animal experiments were approved by the Institutional Animal Care and Use Committees of the Yeungnam University (YUMC-AEC2017–013 and YUMC-ACE2019–033). The acute skeletal muscle injury model was generated by a single intramuscular (left gastrocnemius, 100 μl 10 uM) injection of cardiotoxin (CTX; Najamossambica; Sigma Aldrich, St. Louis, MO, USA). Contralateral right gastrocnemius muscles were injected with phosphate buffered saline and used as controls. To investigate the effects of the CWE of *G. uralensis* or liquiritigenin on muscle regeneration, two-month-old mice were orally administered CWE of *G. uralensis* (100 mg/kg) or liquiritigenin (15 mg/kg) once a day for 9 days from one day before CTX injection. Seven days after CTX injection, mice were anesthetized with avertin i.p. (Sigma Aldrich, a mixture of tert-amyl alcohol and 2,2,2-tribromoethanol) and gastrocnemius muscles were excised for Western blotting and immunohistochemistry analysis.

### 4.5. Cell Culture

Murine C2C12 myoblast cells (Korean Cell Line Bank, Seoul, Korea) were cultured in growth media [DMEM (HyClone Laboratories, South Logan, UT, USA) + 10% FBS (fetal bovine serum, HyClone Laboratories) + 1% P/S (Penicillin/Streptomycin, Thermo Fisher Scientific, Waltham, MA, USA)] in a humidified 5% CO_2_ incubator at 37 °C.

### 4.6. Cell Proliferation Assay

Cells were cultured in growth media supplemented with *G. uralensis* (CWE 50, 100, or 200 µg/mL, performed 6 times), *G. uralensis*-fractions (EX, DCM, EtOAc, BuOH, H_2_O in DMSO, at 25 µg/mL, 10 times), or EtOAc-single compounds (0.5 ng/mL, 14 times) at 37 °C for 1 or 2 days. Non-treated and treated cells were washed with fresh DMEM and then cultured in 0.5 mg/mL of MTT reagent (Sigma-Aldrich, 500 μL MTT Sol./well) at 37 °C for 1 h. After removing the MTT reagent, formazan crystals were dissolved with DMSO (500 μL /well) and absorbances were measured at 540 nm using a spectrophotometer (Tecan Group Ltd., Männedorf, Switzerland). Percentage cell proliferations were calculated by expressing the ODs of treated cells as percentages of non-treated controls.

### 4.7. Cell Differentiation

To induce myogenic differentiation, when cells reached 100% confluence, proliferation media was switched to differentiation media [DMEM+2% FBS+1% P/S for 4 days] supplemented with the CWE of *G. uralensis* (100, µg/mL), *G. uralensis*-fractions (EX, DCM, EtOAc, BuOH, or H_2_O fractions; 25 µg/mL), or EtOAc-single compounds (0.25 ng/mL).

### 4.8. Scratch Experiment

Cells were cultured in DMEM+10% FBS+1% P/S until 100% confluent. Cell layers on plates were then scratched, media was added, and cells were incubated for 1 day. Cell recovery was observed using a light microscope (Nikon, Melville, NY, USA) and cell mobility from scratched part to recovered part was measured and then calculated as cell recovery value (%).

### 4.9. Fusion Index

Differentiated cells were washed with PBS, fixed with a methanol/PBS mixture for 2 min, and stained with Giemsa G250 (Sigma Aldrich). Random images were taken at three different regions using a microscope. Nuclei numbers in myotubes and total numbers of nuclei in cells were calculated in each image. Fusion indices were calculated by expressing numbers of nuclei integrated into myotubes as percentages of total numbers of nuclei. All experiments were performed in triplicate [58,59].

### 4.10. Metabolite Analysis

Cells were cultured in differentiation media supplemented with DMSO (EtOAc was dissolved in DMSO, DMSO was used as a control) or EtOAc for 4 days and concentrations of NH_3_ in cultured media were determined using commercial reagent kits (Glucose Bio HT; Roche Diagnostics, Indianapolis, IN, USA) and a Cedex Bio-analyzer (Roche Diagnostics, Indianapolis, IN, USA).

### 4.11. Real-Time RT-PCR

Trizol reagent (Thermo Fisher Scientific) was used to extract total RNA from cells according to the manufacturer’s instructions. RNA (2 µg) in 20 µL of reaction mixture was used to synthesize 1st strand cDNA using random hexamer and reverse transcriptase at 25 °C for 10 min, 37 °C for 120 min, and 85 °C for 5 min. Two micro liters of cDNA and 10 pmole of gene-specific primers were used to analyze gene expression by real-time RT-PCR, which was performed using a 7500 real-time PCR system and a power SYBR Green PCR Master Mix (Thermo Fisher Scientific). Primer information is provided in Supplementary Table S1.

### 4.12. Western Blot

PBS was used to wash cells, which were then lysed with RIPA buffer supplemented with protease inhibitor cocktail (Thermo Fisher Scientific). Total protein concentrations were measured using the Bradford assay. Proteins (40 µg) were electrophoresed in 10 or 12% SDS-polyacrylamide gel and then transferred to PVDF membranes (EMS–Millipore, Billerica, MA, USA). Blots were blocked using 3% skim milk or BSA in Tris-buffered saline (TBS)-Tween 20 for 1 h and then incubated overnight with protein-specific primary antibodies [MYOD (1:500), MYOG (1:500), MuRF1 (1:500), Atrogin1 (1:500), MSTN (1:500), β-actin (1:2000) or GAPDH (1:1000) antibodies (Santa Cruz Biotechnology, Santa Cruz, CA, USA), MYL2 (1:1000, Abcam, MA, USA) or nitrotyrosine (1:500,Young-In Frontier, Seoul, Korea) antibodies] in 1% skim milk or BSA in TBS at 4 °C. After washing with TBST, blots were incubated with horse radish peroxidase (HRP)-conjugated secondary antibody (Santa Cruz Biotechnology) for 1 h at room temperature and then developed with Super Signal West Pico Chemiluminescent Substrate (Thermo Fisher Scientific).

### 4.13. Immunohistochemistry

Muscle tissues were routinely fixed with 10% formalin and embedded in paraffin wax [60]. Paraffin-embedded tissue sections were deparaffinized, hydrated, and endogenous peroxidase was quenched using xylene (Junsei, Tokyo, Japan), an ethanol gradient, and methanol/H_2_O_2_ (Junsei), respectively. After blocking with 1% normal goat serum, sections were incubated with primary antibody [Pax7 (1:50), MYOD (1:50), MYOG (1:50), MYL2 (1:50), MSTN (1:50), MuRF1 (1:50), Atrogin1(1:50), nitrotyrosine (1:50)] overnight at 4 °C, and then treated with HRP-conjugated secondary antibody (1:100; Santa Cruz Biotechnology) at room temperature for 1 h. Sections were then counterstained with hematoxylin, dehydrated, mounted, and examined under a light microscope (Leica, Seoul, Korea). After Hematoxylin and Eosin staining (H&E, Thermo Fisher Scientific), muscle tissues were examined for morphological changes under a light microscope.

### 4.14. Analysis of Muscle Mass Reduction and Muscle Fiber Diameters

The reduction ratio of muscle mass (%) was calculated using a given formula:

Reduction ratio of muscle mass (%) = 100 − (CTX injected gastrocnemius muscle/non-injected gastrocnemius muscle) × 100.(1)

Muscle tissue sections were H&E stained, and images were captured under a light microscope. Muscle fiber diameters were measured using ImageJ software [61].

### 4.15. FAF-Drugs4 Analysis

The ADMET (absorption, distribution, metabolism, elimination, and toxicity) pharmacokinetic properties of compounds were analyzed using FAFdrugs4 (https://fafdrugs4.rpbs.univ-paris-diderot.fr/), an open-access tool for the prediction of ADMET properties that allows filtering according to Egan’s rule, Lipinski’s rule of five, and Veber’s rule for the prediction of bioavailabilities, and according to GSK and Pfizer’s rules for overall toxicity predictions [62,63].

### 4.16. Statistical Analysis

Tukey’s Studentized Range test (honest significance difference) was employed to analyze normalized expression mean values to categorize significant gene expression changes. Multi Gauge 3.0 software (Fujifilm, Tokyo, Japan) was used to quantify band intensities in Western blots. Gene and protein expressions were normalized versus GAPDH (the internal control), and the analysis was conducted using one-way ANOVA and PROC GLM in SAS, ver.9.0 (SAS Institute, Cary, NC, USA).

## 5. Conclusions

In summary, our results demonstrate that *G. uralensis* promotes the proliferation and differentiation of myoblasts and muscle regeneration by upregulating the expressions of the myogenic marker genes (MYOD, MYOG, and MYL2) and by downregulating the expression of MSTN (a muscle growth inhibitor). Based on the observed inhibitory potency of *G. uralensis* against atrophy and protein degradation-related marker genes (Atrogin1 and MuRF1), we suggest that *G. uralensis* be considered a therapeutic agent for the prevention and treatment of muscular dystrophy, sarcopenia, and cachexia. In addition, the observed anti-inflammatory effect of *G. uralensis* suggests therapeutic applications in the context of inflammatory disease.

## Figures and Tables

**Table 1:** Compounds in the *G. uralensis*–EtOAc fraction.

Isolated Compounds	Weight (mg)	Yield (%) ^a^	NMR Spectra	
			^1^H	^13^C
**4**-hydroxybenzoic acid	23.90	0.0006	600 MHz	150 MHz
Liquiritigenin	108.5	0.0026	600 MHz	150 MHz
*R*-(-)-Vestitol	51.70	0.0012	600 MHz	150 MHz
Isoliquiritigenin	145.5	0.0035	600 MHz	150 MHz
Medicarpin	11.20	0.0003	600 MHz	150 MHz
Tetrahydroxymethoxychalcone	35.20	0.0008	600 MHz	150 MHz
Licochalcone B	5.600	0.0001	600 MHz	150 MHz
Liquiritin	969.0	0.0231	600 MHz	150 MHz
Liquiritin apioside	113.5	0.0027	600 MHz	150 MHz
Ononin	22.20	0.0005	600 MHz	150 MHz

The structures of these compounds were determined by NMR. ^a^ Percentage of isolated compound yield (g/g plant dry weight) extracted from *G. uralensis*.

## Data Availability

The data presented in this study are available in this article and the accompanying supplementary material.

## Supplementary Materials

The following are available online at https://www.mdpi.com/1422-0067/22/2/876/s1, Supplementary Table S1. Primer information; Supplementary Figure S1. C2C12 cells were cultured in growth media supplemented with *G. uralensis* for 1 day. Ki67, CyclinA2, and MSTN expressions were analyzed by real time RT-PCR; Supplementary Figure S2. *G. uralensis* fractionation procedure (red square box: fractions bioassayed); Supplementary Figure S3. Metabolite analysis after culture in media supplemented with DMSO (as real control for comparison with EtOAc) or the EtOAc fraction. Cells were cultured in differentiation media supplemented with DMSO or DMSO+ EtOAc fraction of *G. uralensis* CWE for 2 days. Cultured media were then collected and NH_3_ concentrations were analyzed in DMSO or EtOAc fraction treated cells. Control indicates fresh differentiation media. * *p* ≤ 0.05, ** *p* ≤ 0.001, *** *p* ≤ 0.0001; Supplementary Figure S4. ^1^H NMR spectrum of compound 1 in MeOD-*d*_4_ (600 MHz); Supplementary Figure S5. ^13^C NMR spectrum of compound 1 in MeOD-*d*_4_ (150 MHz); Supplementary Figure S6. ^1^H NMR spectrum of compound 2 in MeOD-*d*_4_ (600 MHz); Supplementary Figure S7. ^13^C NMR spectrum of compound 2 in MeOD-*d*_4_ (150 MHz); Supplementary Figure S8. ^1^H NMR spectrum of compound 3 in Acetone-*d_6_* (600 MHz); Supplementary Figure S9. ^13^C NMR spectrum of compound 3 in Acetone-*d_6_* (150 MHz); Supplementary Figure S10. ^1^H NMR spectrum of compound 4 in MeOD-*d*_4_ (600 MHz); Supplementary Figure S11. ^13^C NMR spectrum of compound 4 in MeOD-*d*_4_ (150 MHz); Supplementary Figure S12. ^1^H NMR spectrum of compound 5 in CDCl_3_ (600 MHz); Supplementary Figure S13. ^13^C NMR spectrum of compound 5 in CDCl_3_ (150 MHz); Supplementary Figure S14. ^1^H NMR spectrum of compound 6 in MeOD-*d*_4_ (600 MHz); Supplementary Figure S15. ^13^C NMR spectrum of compound 6 in MeOD-*d*_4_ (150 MHz); Supplementary Figure S16. ^1^H NMR spectrum of compound 7 in MeOD-*d*_4_ (600 MHz); Supplementary Figure S17. ^13^C NMR spectrum of compound 7 in MeOD-*d*_4_ (150 MHz); Supplementary Figure S18. 1H NMR spectrum of compound 8 in DMSO-*d_6_* (600 MHz); Supplementary Figure S19. ^13^C NMR spectrum of compound 8 in DMSO-*d_6_* (150 MHz); Supplementary Figure S20. ^1^H NMR spectrum of compound 9 in MeOD-*d*_4_ (600 MHz); Supplementary Figure S21. ^13^C NMR spectrum of compound 9 in MeOD-*d*_4_ (150 MHz); Supplementary Figure S22. ^1^H NMR spectrum of compound 10 in DMSO-*d_6_* (600 MHz); Supplementary Figure S23. ^13^C NMR spectrum of compound 10 in DMSO-*d_6_* (150 MHz).
