# Supplementary material for: Isolation and Characterization of Compounds from Glycyrrhiza uralensis as Therapeutic Agents for the Muscle Disorders
Source: Int J Mol Sci. 2021 Jan 16;22(2):876. doi: 10.3390/ijms22020876 (PMC7830955; doi:10.3390/ijms22020876)

Supplementary Table S1. Primer information

Species	Gene	Product size (bp)	Tm (°C)	Sequence (F)	Sequence (R)
Mouse	GAPDH	155	59	5'-tgctggtgctgagtatgtcg-3'	5'-caagcagttggtggtacagg-3'
Mouse	MYOD	213	59	5'-aggagcacgcacacttctct-3'	5'-tctcgaaggcctcattcact-3'
Mouse	MYOG	185	59	5'-tccagtacattgagcgccta-3'	5'-caaatgatctcctgggttgg-3'
Mouse	MYL2	177	59	5'-aaagaggctccaggtccaat-3'	5'-cctctctgcttgtgtggtca-3'
Mouse	Atrogin1	160	59	5'-ttcagcagcctgaactacga-3'	5'-tgaaagcttcccccaaagta-3'
Mouse	MuRF1	206	59	5'-tgaggtgcctacttgctcct-3'	5'-tcacctggtggctattctcc-3'
Mouse	MSTN	163	59	5'-acgctaccacggaaacaatc-3'	5'-ggagtcttgacgggtctgag-3'
Mouse	CyclinA2	227	59	5'-ctgtctctttacccggagca-3'	5'-Agtgatgtctggctgcctct-3'
Mouse	Ki67	199	59	5'-gggcgaagttcacagtcaat-3'	5'-ctccttcactggggtcttga-3'

Supplementary Figure S1. Ki67, CyclinA2, and MSTN expression with *G. uralensis* crude water extract treatment.

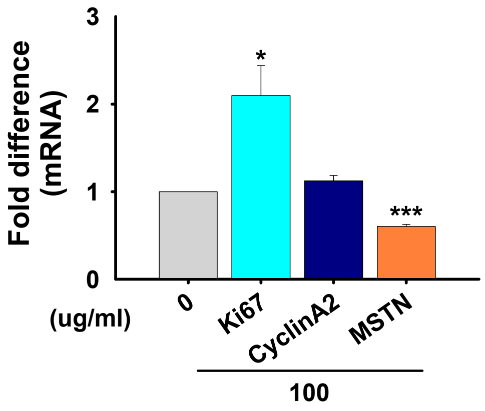

Supplementary Figure S2. Procedure of *G. uralensis* fraction.

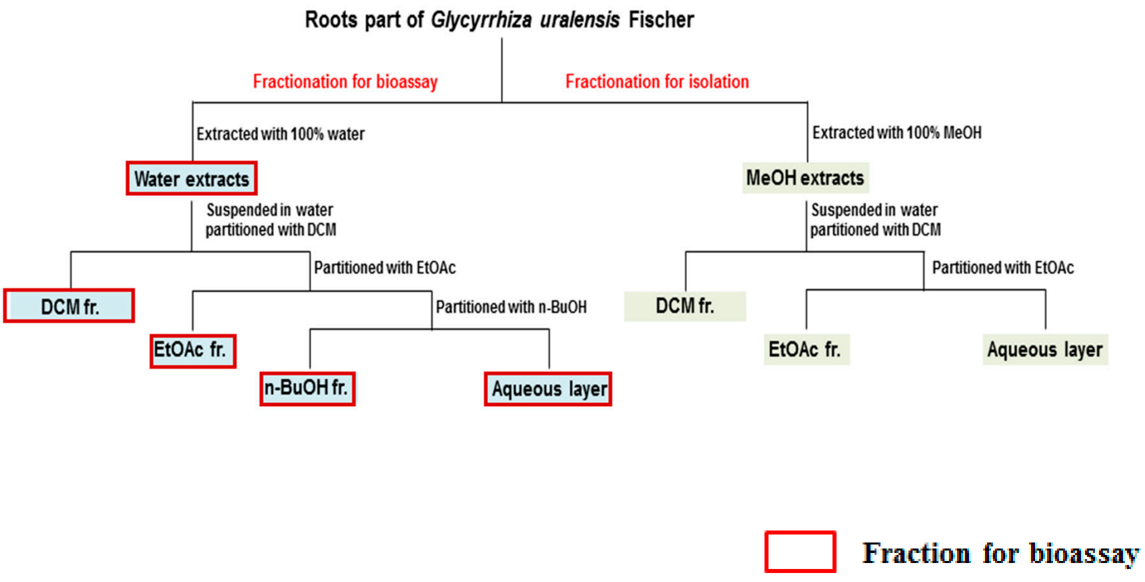

Supplementary Figure S3. Metabolite (NH_3) analysis in cultured media supplemented with EtOAc fraction

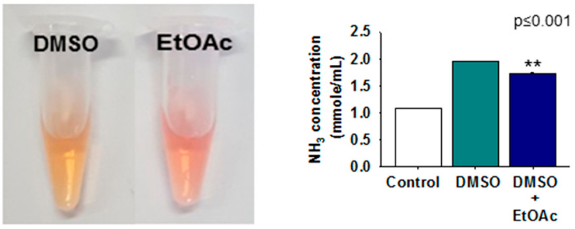

Supplementary Figure S4. ^{1}H NMR spectrum of compound **1** in MeOD-d_4 (600 MHz)

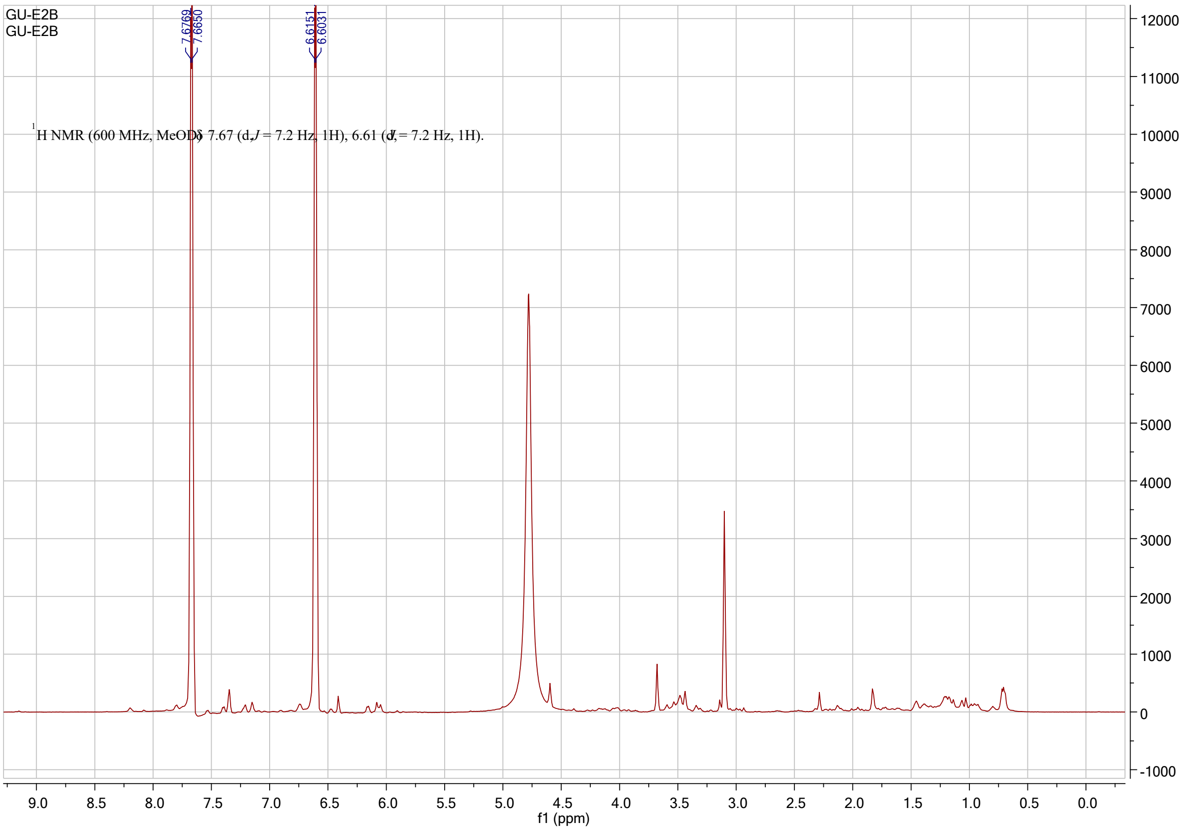

Supplementary Figure S5. ^{13}C NMR spectrum of compound **1** in MeOD-d_4 (150 MHz)

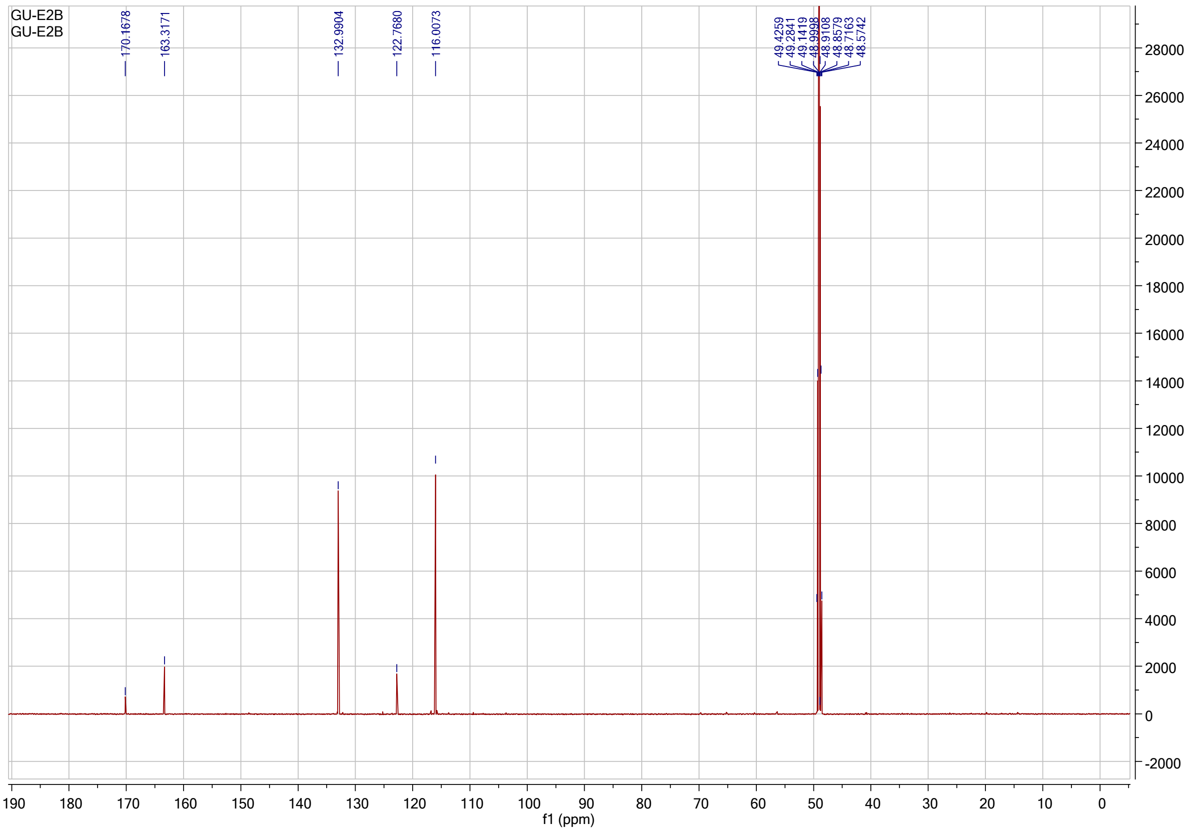

Supplementary Figure S6. ^{1}H NMR spectrum of compound **2** in MeOD-*d*$_4$ (600 MHz)

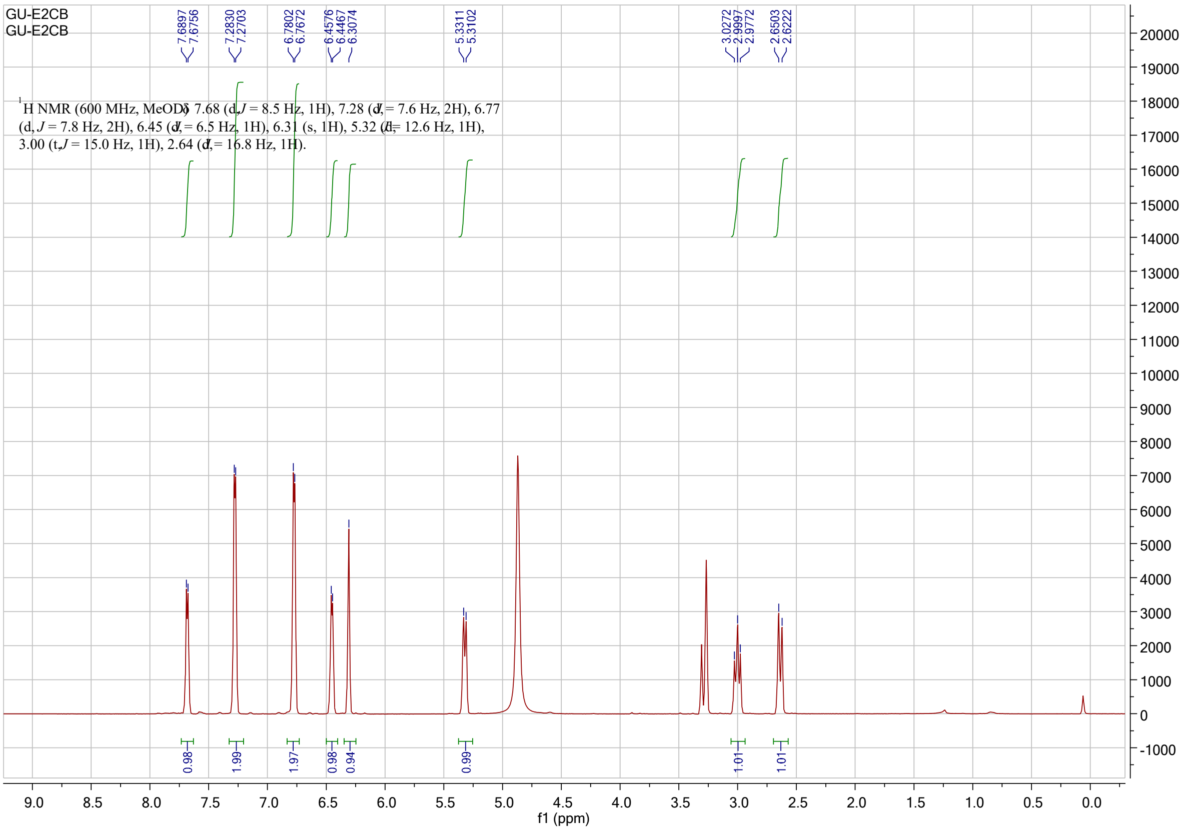

Supplementary Figure S7. ^{13}C NMR spectrum of compound **2** in MeOD-d_4 (150 MHz)

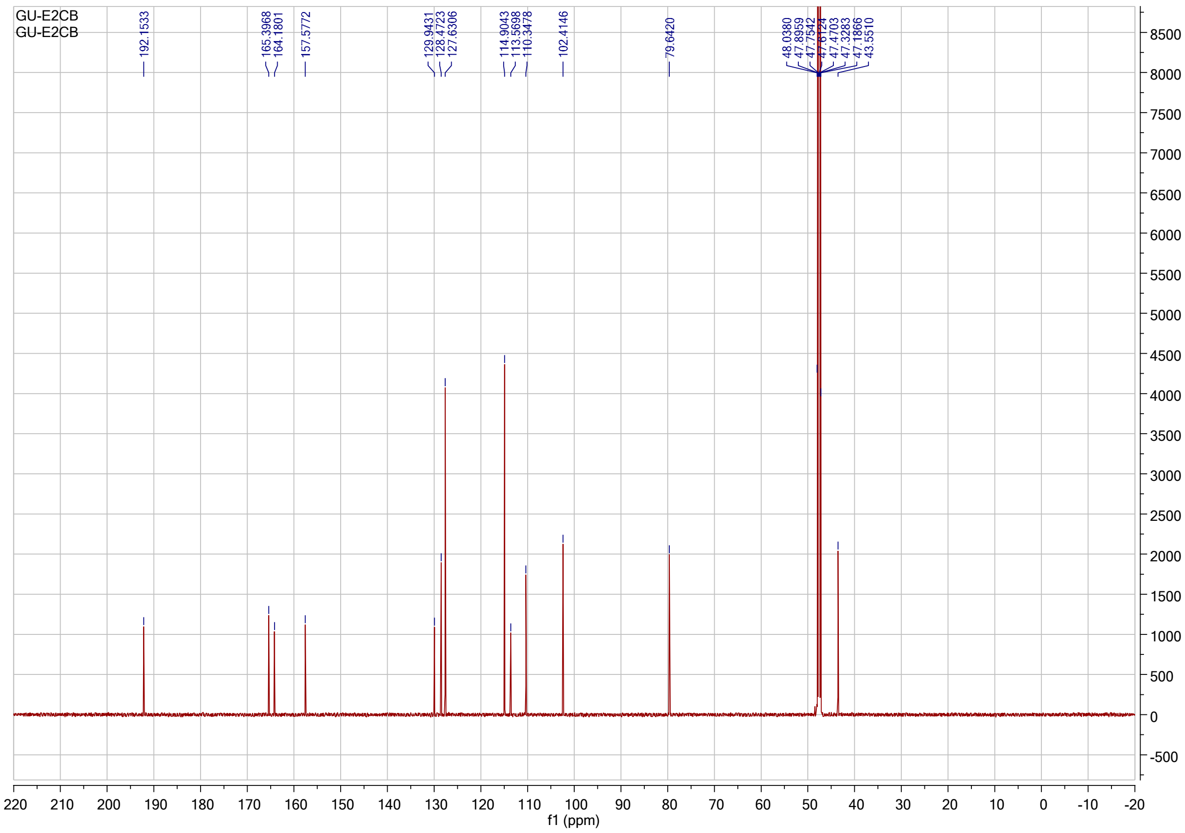

Supplementary Figure S8. ^{1}H NMR spectrum of compound **3** in Acetone-*d₆* (600 MHz)

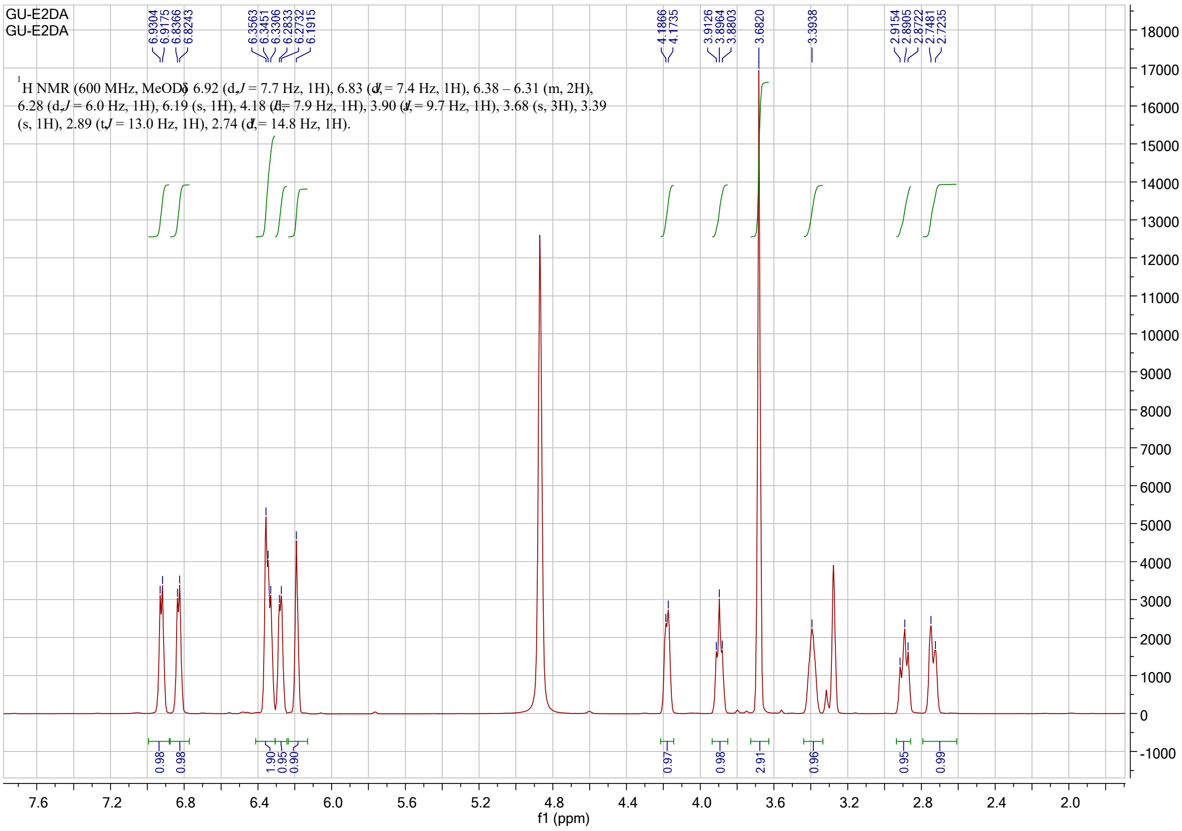

Supplementary Figure S9. ^{13}C NMR spectrum of compound **3** in Acetone-d_6 (150 MHz)

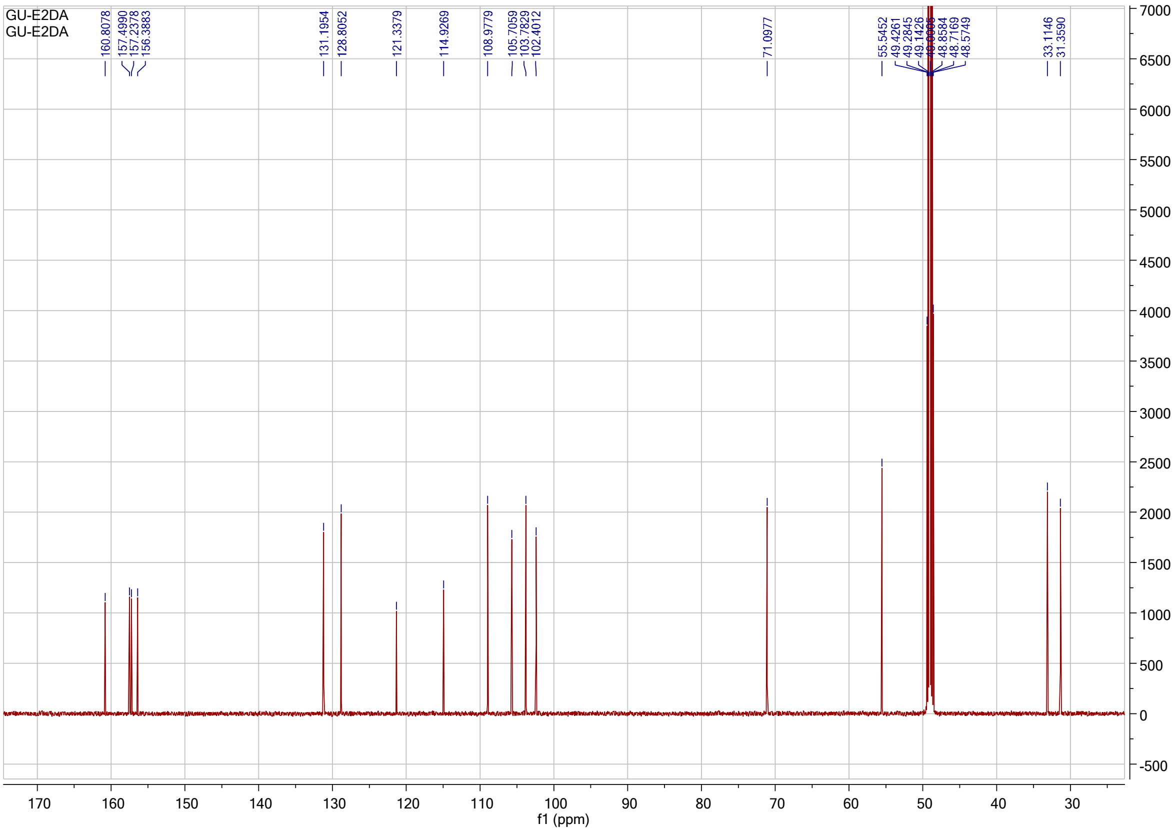

Supplementary Figure S10. ^{1}H NMR spectrum of compound **4** in MeOD-d_4 (600 MHz)

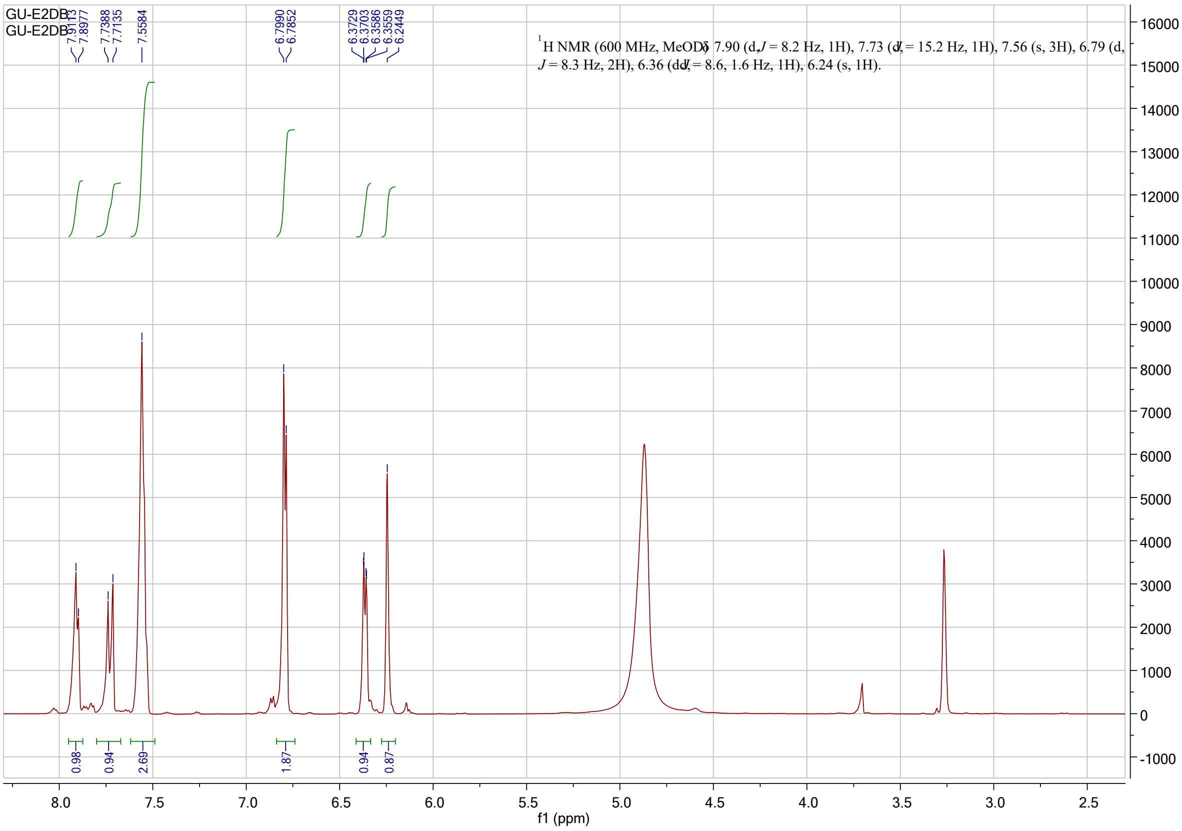

Supplementary Figure S11. ^{13}C NMR spectrum of compound **4** in MeOD-d_4 (150 MHz)

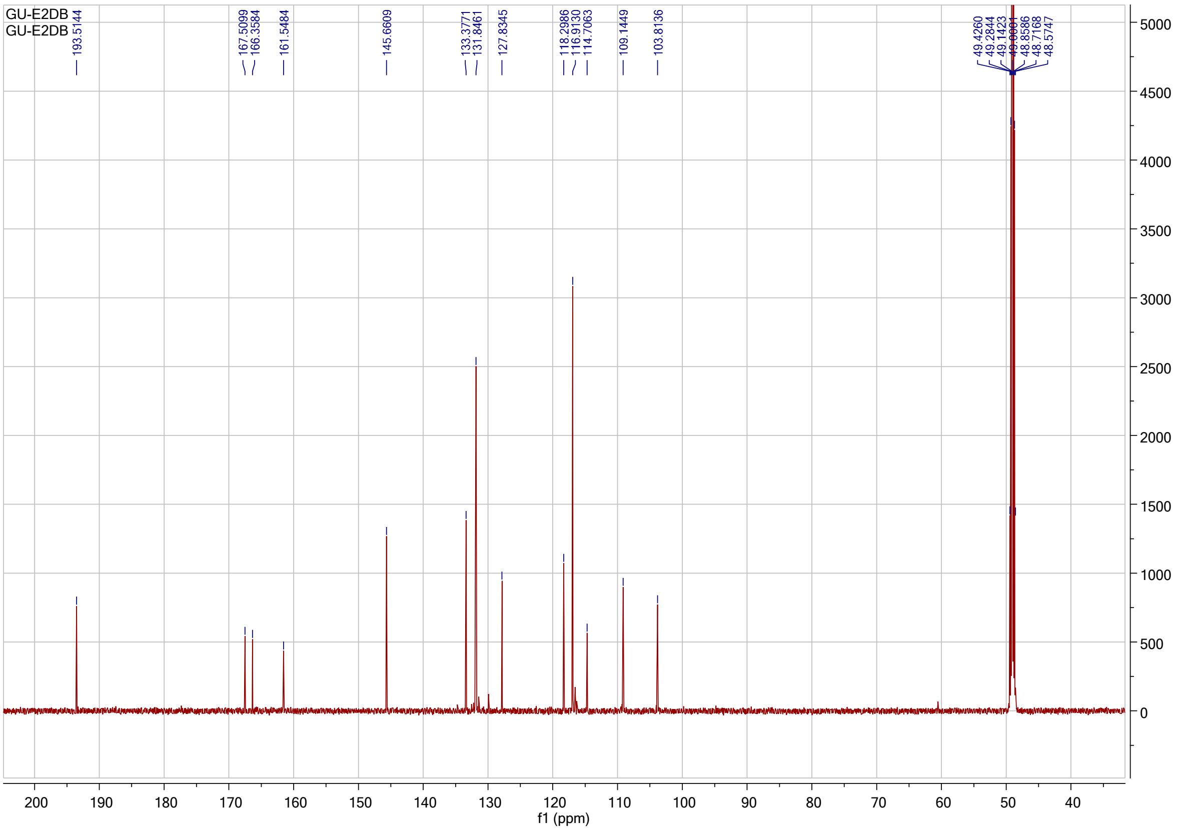

Supplementary Figure S12. ^{1}H NMR spectrum of compound **5** in $CDCl_3$ (600 MHz)

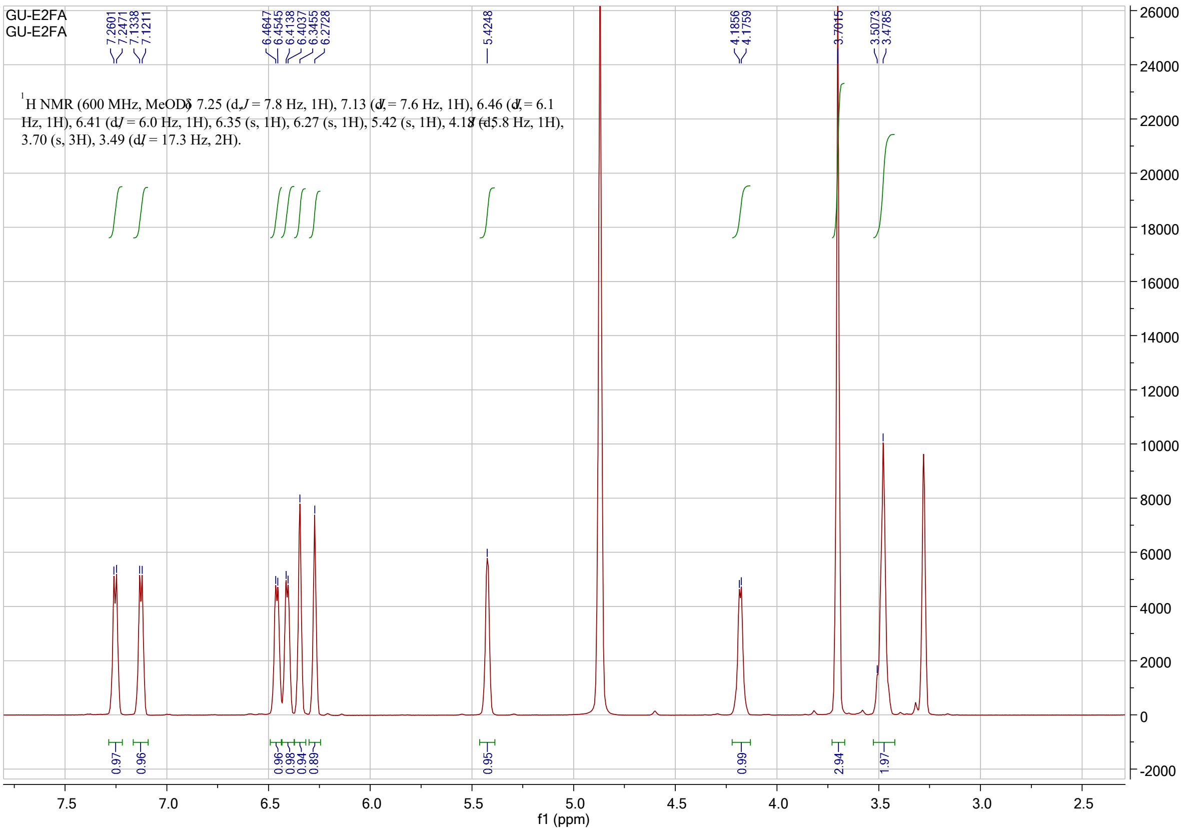

Supplementary Figure S13. ^{13}C NMR spectrum of compound **5** in $CDCl_3$ (150 MHz)

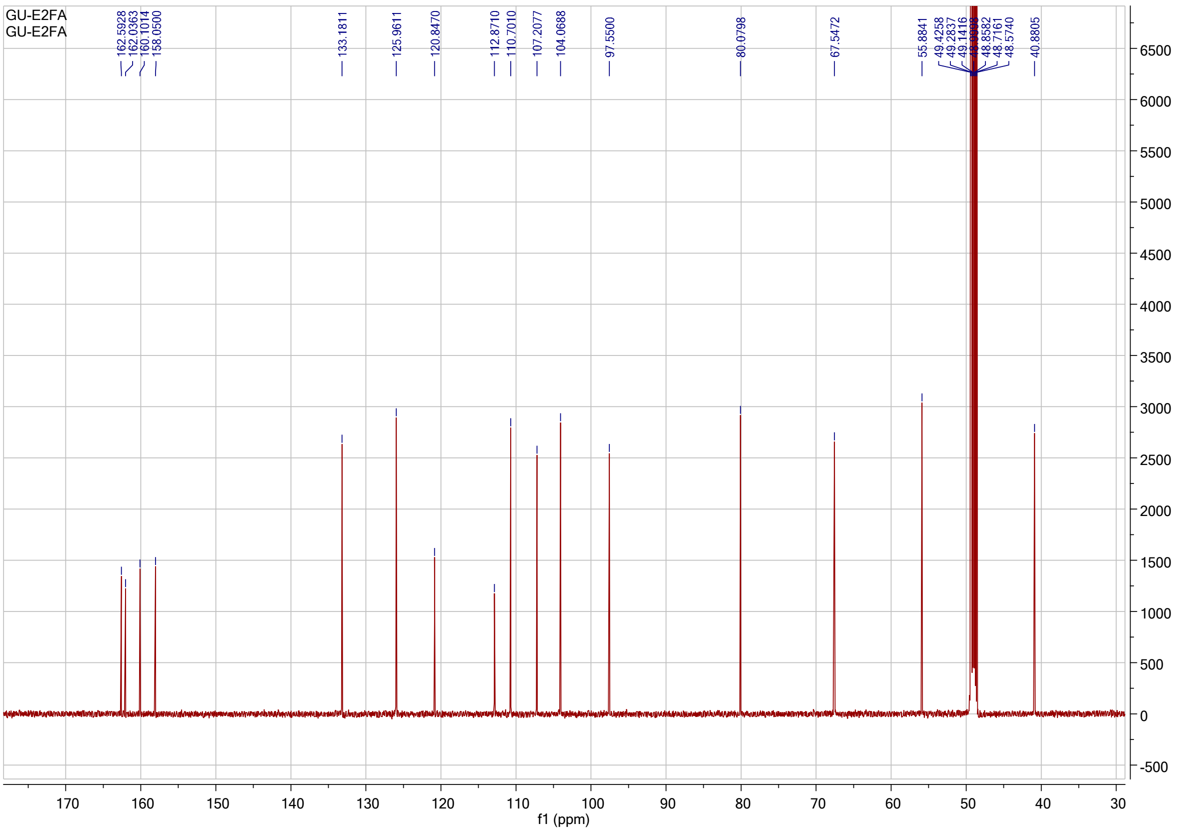

Supplementary Figure S14. ^{1}H NMR spectrum of compound **6** in MeOD-d_4 (600 MHz)

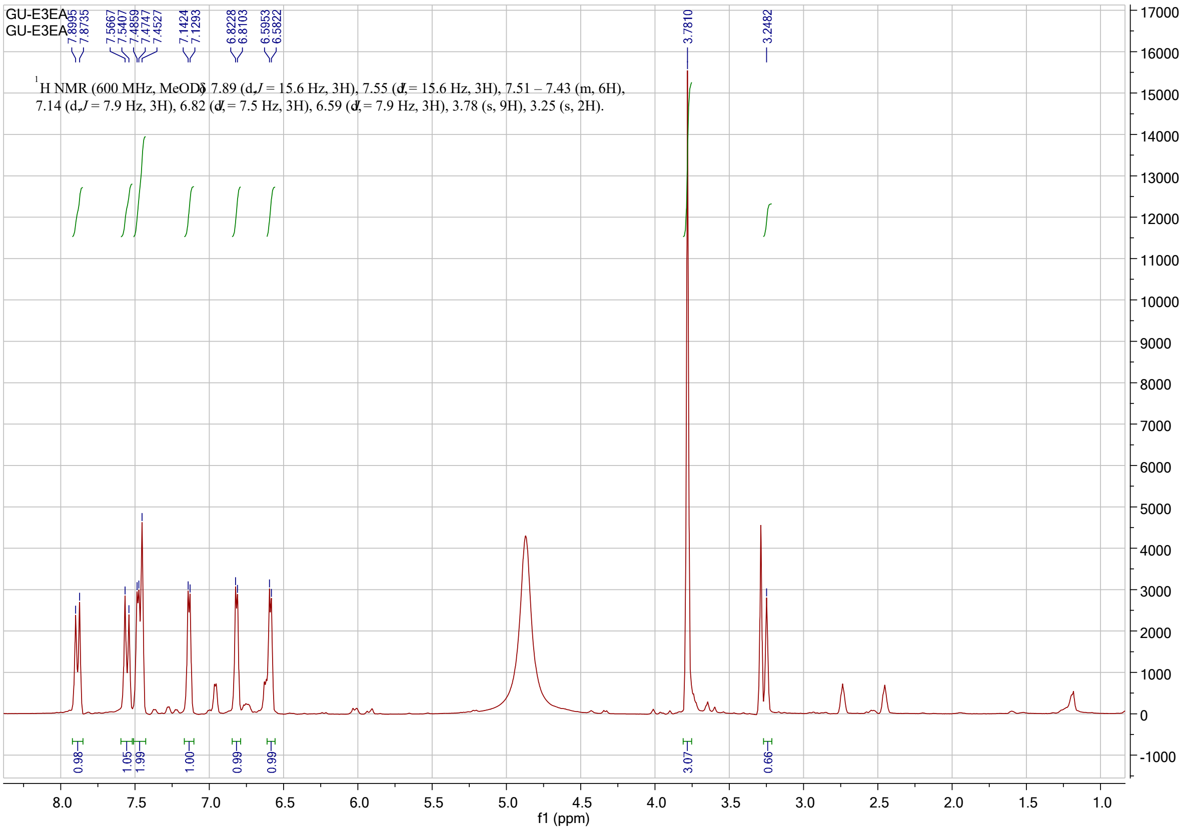

Supplementary Figure S15. ^{13}C NMR spectrum of compound **6** in MeOD-d_4 (150 MHz)

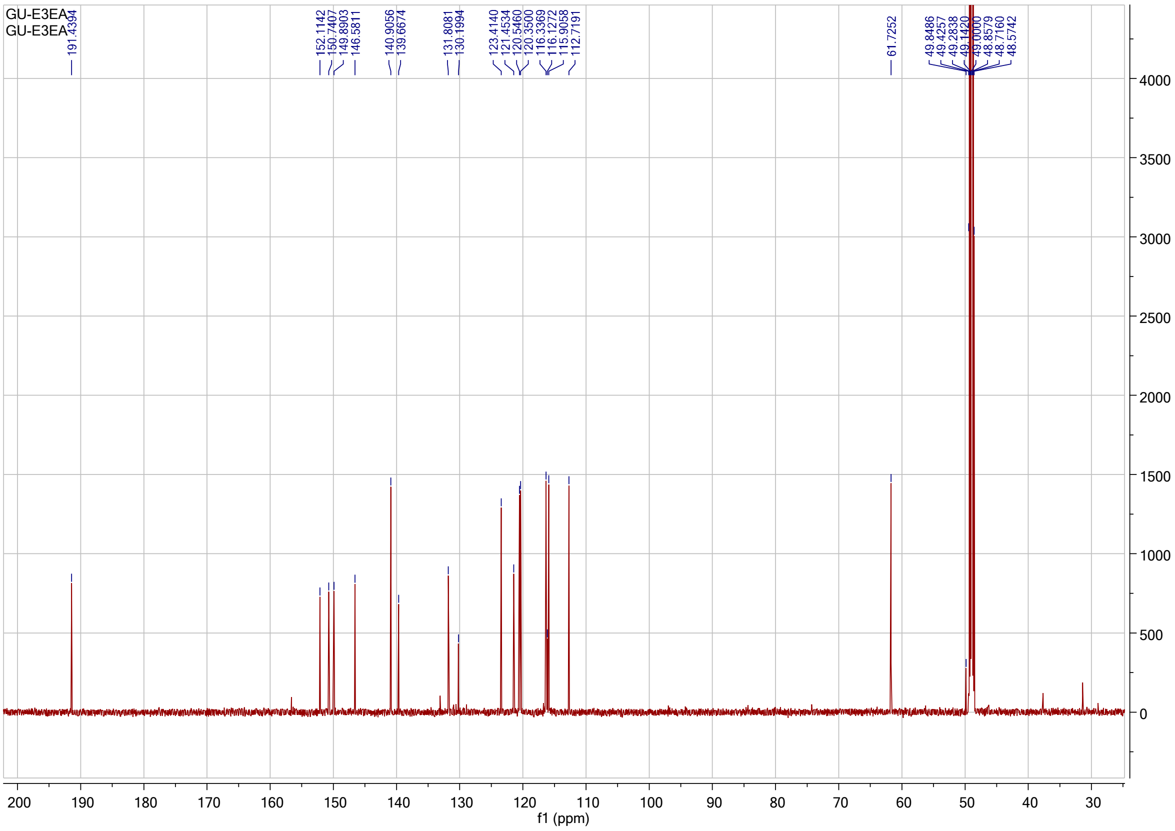

Supplementary Figure S16. ^{1}H NMR spectrum of compound **7** in MeOD-d_4 (600 MHz)

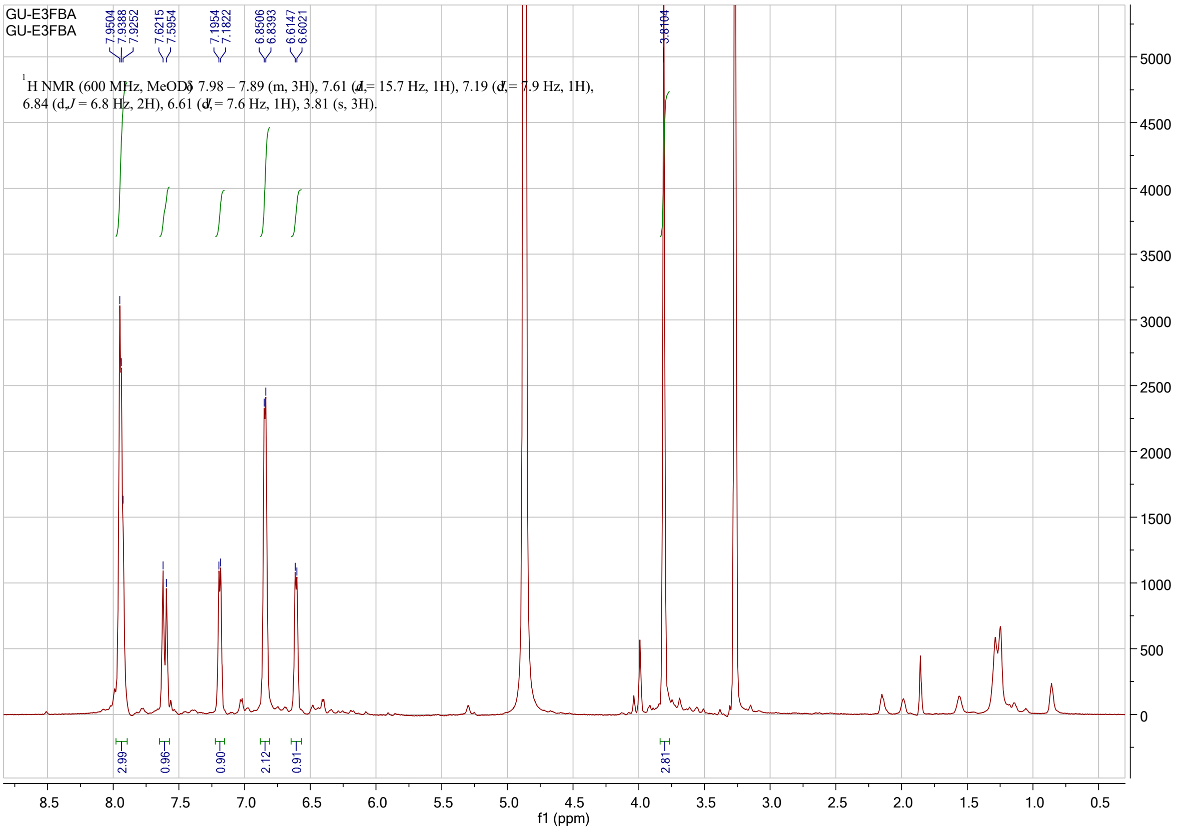

Supplementary Figure S17. ^{13}C NMR spectrum of compound **7** in MeOD-d_4 (150 MHz)

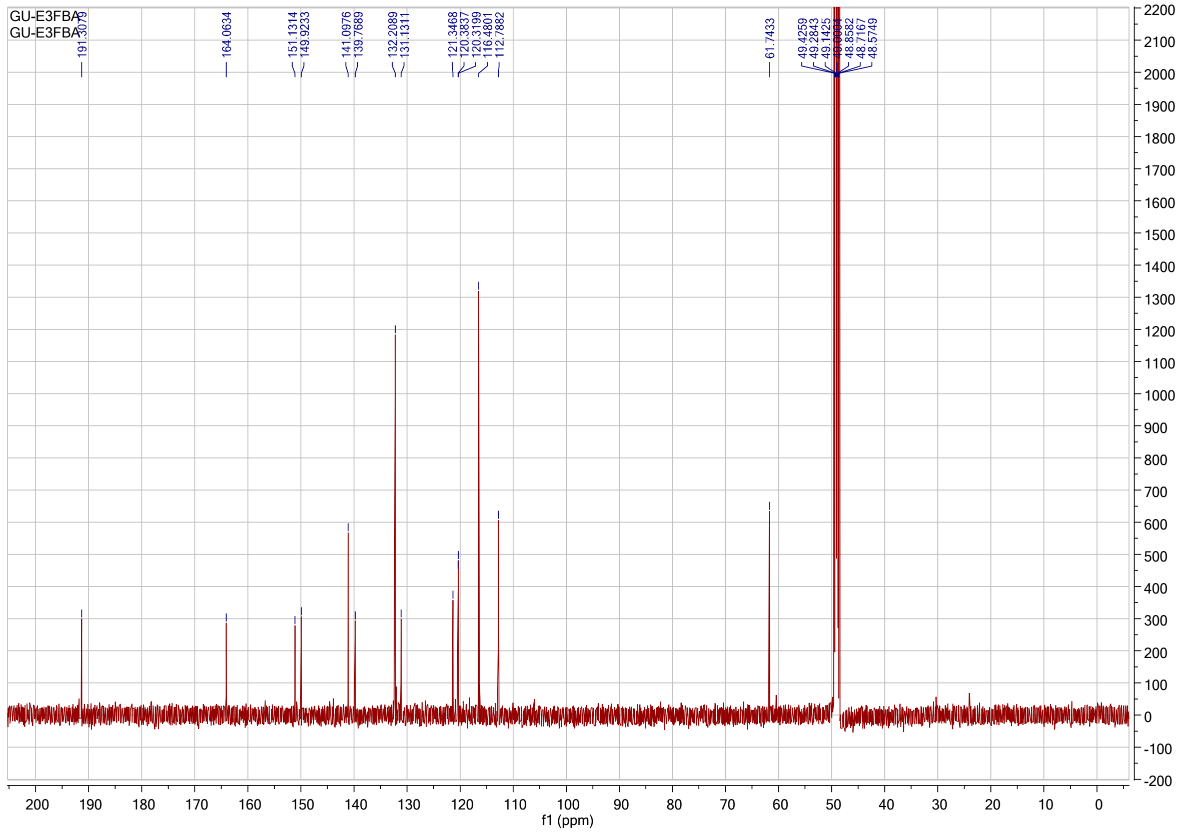

Supplementary Figure S18. ^{1}H NMR spectrum of compound **8** in DMSO-d_6 (600 MHz)

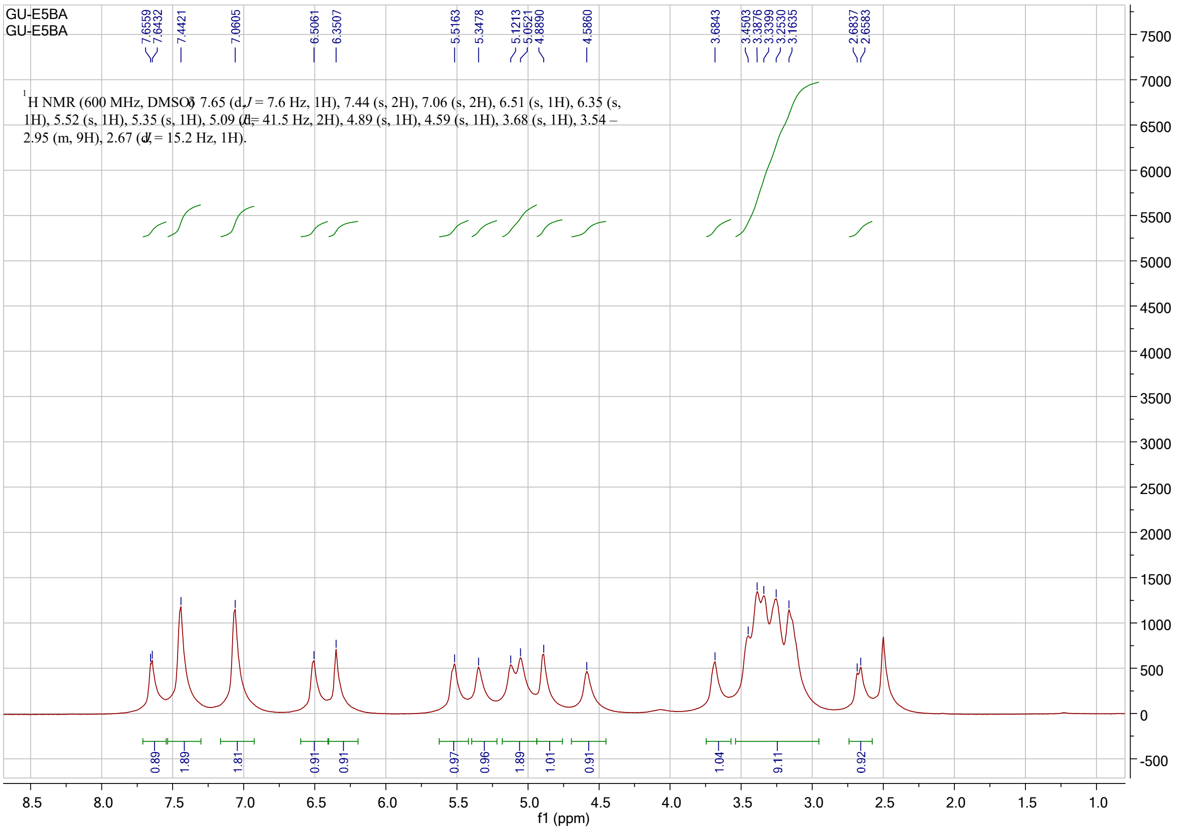

Supplementary Figure S19. ^{13}C NMR spectrum of compound **8** in DMSO-d_6 (150 MHz)

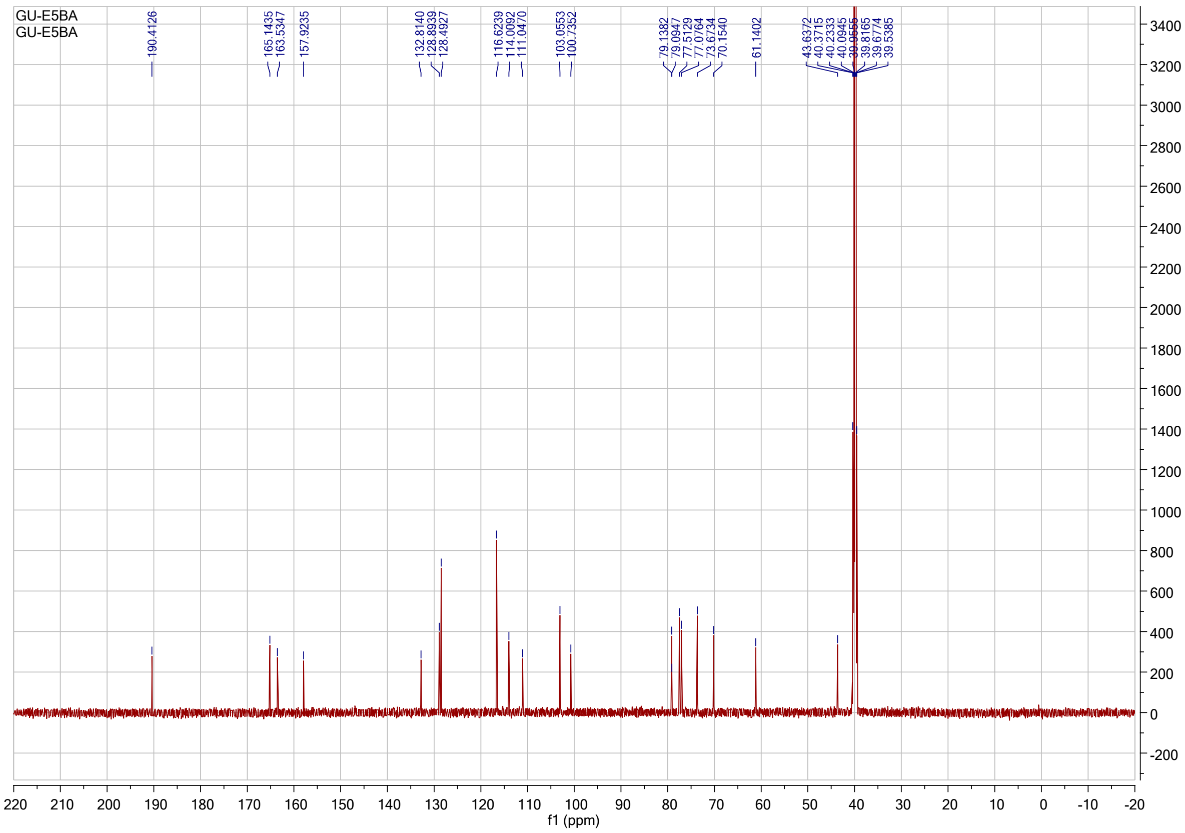

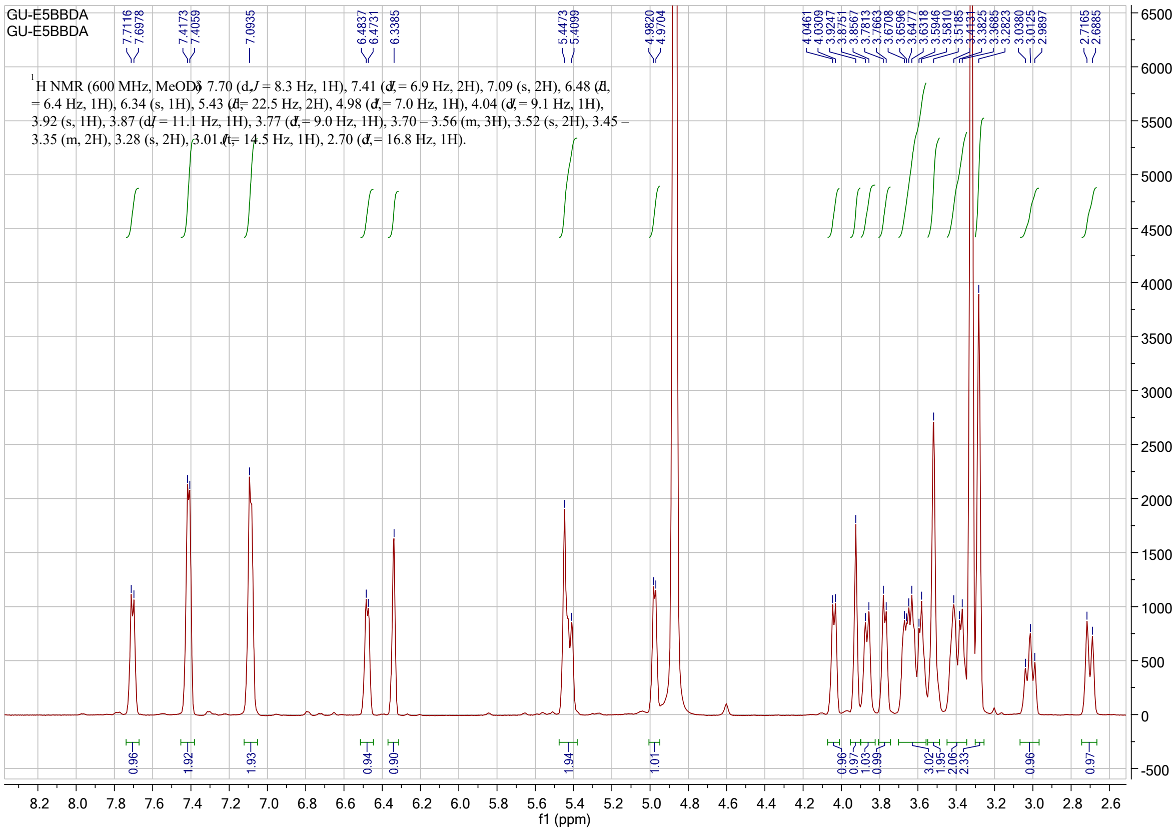

Supplementary Figure S20. ^{1}H NMR spectrum of compound **9** in MeOD-d_4 (600 MHz)

Supplementary Figure S21. ^{13}C NMR spectrum of compound **9** in MeOD-d_4 (150 MHz)

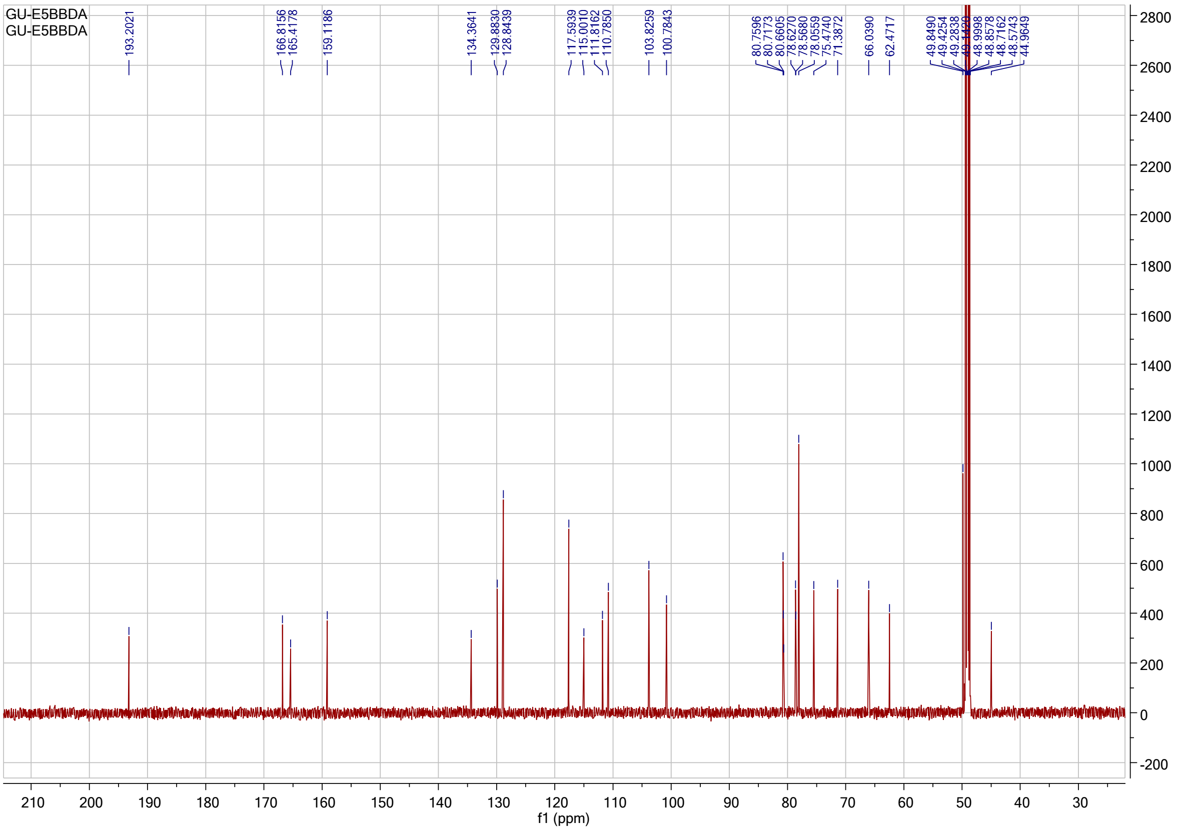

Supplementary Figure S22. ^{1}H NMR spectrum of compound **10** in DMSO-d_6 (600 MHz)

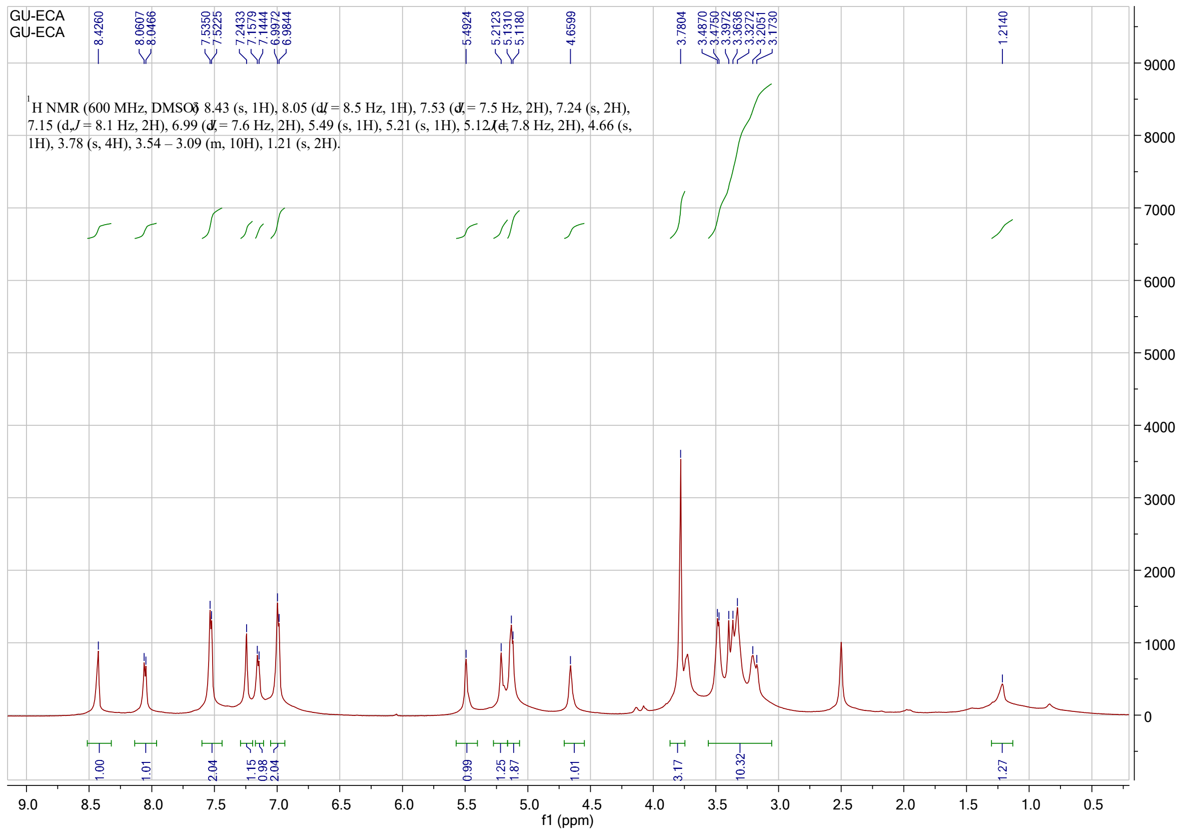

Supplementary Figure S23. ^{13}C NMR spectrum of compound **10** in DMSO-*d*$_6$ (150 MHz)

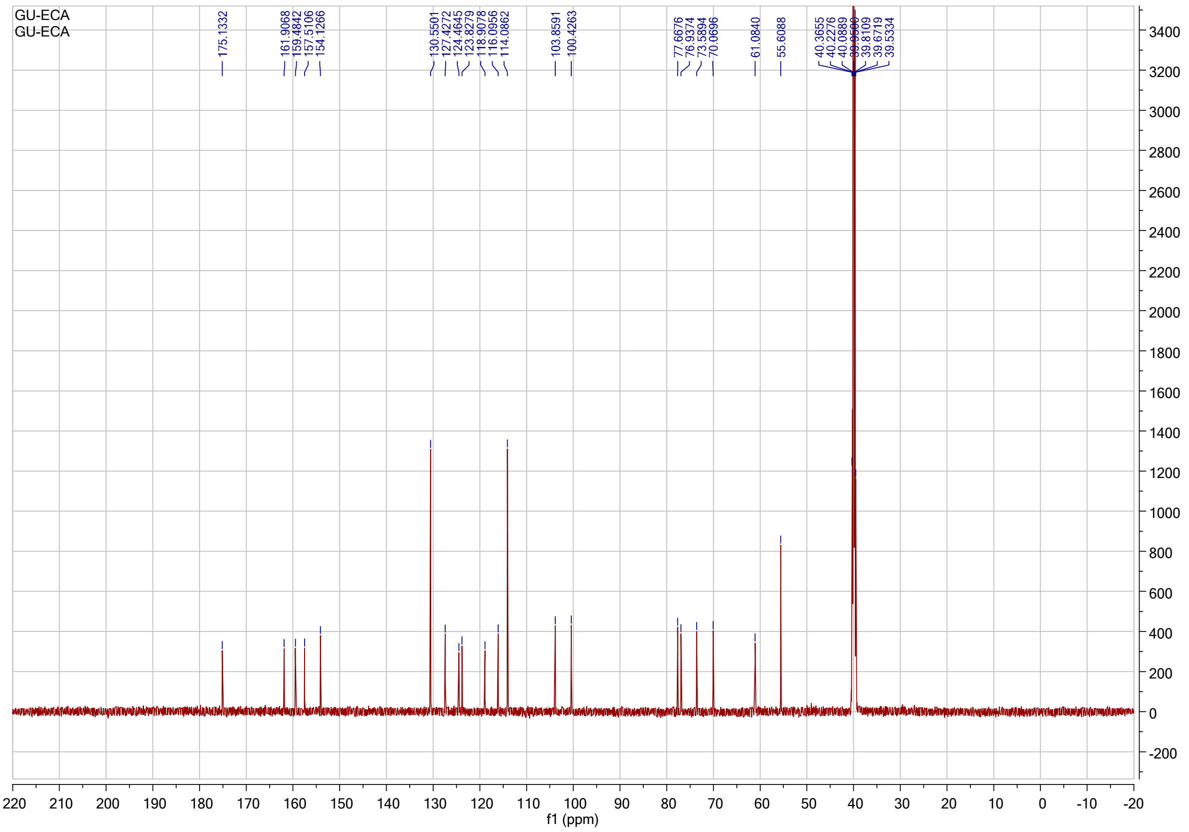

Supplement: Supplementary file 1 [file ijms-22-00876-s001.pdf]
